# Sequence-based prediction of permissive stretches for internal protein tagging and knockdown

**DOI:** 10.1186/s12915-017-0440-0

**Published:** 2017-10-30

**Authors:** Sabine Oesterle, Tania Michelle Roberts, Lukas Andreas Widmer, Harun Mustafa, Sven Panke, Sonja Billerbeck

**Affiliations:** 1 Department of Biosystems Science and Engineering, ETH Zürich, Mattenstrasse 26, 4058 Basel, Switzerland; 2 Swiss Institute of Bioinformatics, Mattenstrasse 26, 4058 Basel, Switzerland; 3 Life Science Zürich Graduate School in Systems Biology, Zürich, Switzerland; 4 Department of Computer Science, ETH Zürich, Zürich, Switzerland; 5 Present address: Chemistry Department, Columbia University, 550 West 120th Street, New York, NY 10027 USA

**Keywords:** Permissive site, Internal protein tagging, TEV protease, Protein knockdowns, Cell-free biotechnology

## Abstract

**Background:**

Internal tagging of proteins by inserting small functional peptides into surface accessible permissive sites has proven to be an indispensable tool for basic and applied science. Permissive sites are typically identified by transposon mutagenesis on a case-by-case basis, limiting scalability and their exploitation as a system-wide protein engineering tool.

**Methods:**

We developed an apporach for predicting permissive stretches (PSs) in proteins based on the identification of length-variable regions (regions containing indels) in homologous proteins.

**Results:**

We verify that a protein's primary structure information alone is sufficient to identify PSs. Identified PSs are predicted to be predominantly surface accessible; hence, the position of inserted peptides is likely suitable for diverse applications. We demonstrate the viability of this approach by inserting a Tobacco etch virus protease recognition site (TEV-tag) into several PSs in a wide range of proteins, from small monomeric enzymes (adenylate kinase) to large multi-subunit molecular machines (ATP synthase) and verify their functionality after insertion. We apply this method to engineer conditional protein knockdowns directly in the *Escherichia coli* chromosome and generate a cell-free platform with enhanced nucleotide stability.

**Conclusions:**

Functional internally tagged proteins can be rationally designed and directly chromosomally implemented. Critical for the successful design of protein knockdowns was the incorporation of surface accessibility and secondary structure predictions, as well as the design of an improved TEV-tag that enables efficient hydrolysis when inserted into the middle of a protein. This versatile and portable approach can likely be adapted for other applications, and broadly adopted. We provide guidelines for the design of internally tagged proteins in order to empower scientists with little or no protein engineering expertise to internally tag their target proteins.

**Electronic supplementary material:**

The online version of this article (doi:10.1186/s12915-017-0440-0) contains supplementary material, which is available to authorized users.

## Background

Small functional peptides have shifted into focus as tools for advanced in vivo imaging and chemical biology. Peptides offer a diversity of functions condensed into few amino acids: they can serve as highly specific binding motifs for small molecules or metals [1–3] and as recognition sequences for proteases [4] or labelling enzymes [5, 6], and they can self-catalyse covalent bonding [7]. Furthermore, they can mimic carbohydrates [8] and serve as inhibitors [9] or as epitopes able to elicit an immune response [10] or modulate innate immunity [11]. Tagging proteins with functional peptides has revolutionised the ease and scale of protein purification, opened novel pathways for vaccine design, and also enabled visualisation and characterisation of biological systems and processes in vivo and in vitro [12]. Although many proteins can be tagged N- or C-terminally, it is frequently necessary or desirable to insert a tag internally at a permissive site that accepts additional amino acids. There are several possible reasons for doing this, in addition to multiple tagging. The termini of a protein may be functionally relevant or buried [13–16] so that an internal tag might be more resistant to proteolytic degradation than a terminal fusion [15], the inserted peptide may need to be structurally stabilised in order to exhibit its function (e.g. sufficiently rigidified as shown for lanthanide binding tags for nuclear magnetic resonance (NMR) studies [3]), or the specific peptide’s conferred function may require it to be located internally (as is the case for engineering conditional protein knockdowns by protease hydrolysis site insertion) [13, 17–19].

Due to the limited understanding of the precise mechanisms underlying site permissiveness, the full potential of internal protein tagging has largely remained untapped. State-of-the-art approaches are based on transposon mutagenesis [14, 18, 19]. We previously showed the feasibility of this approach by identifying permissive sites in the molecular chaperonin GroEL [13]. However, transposon mutagenesis is laborious, involving several in vitro DNA manipulation and engineering steps. This limits its potential use for high-throughput protein tagging and thus prevents the true exploitation of permissive sites as a proteome-wide engineering approach. Furthermore, tags inserted by transposon mutagenesis contain large (~19 bp) transposase recognition sites flanking the tag sequence, which we have previously observed to impair protein function [13].

In contrast, rational approaches could minimise the number of engineering cycles for designing and implementing desired functions and thus improve scalability. In combination with novel precision genome-editing tools [20, 21], rational design approaches could augment systematic internal protein tagging efforts directly in the genome, e.g. as recently proposed for systematic protein quantification using designed peptide tags for mass spectrometry [22].

**Fig. 1:**
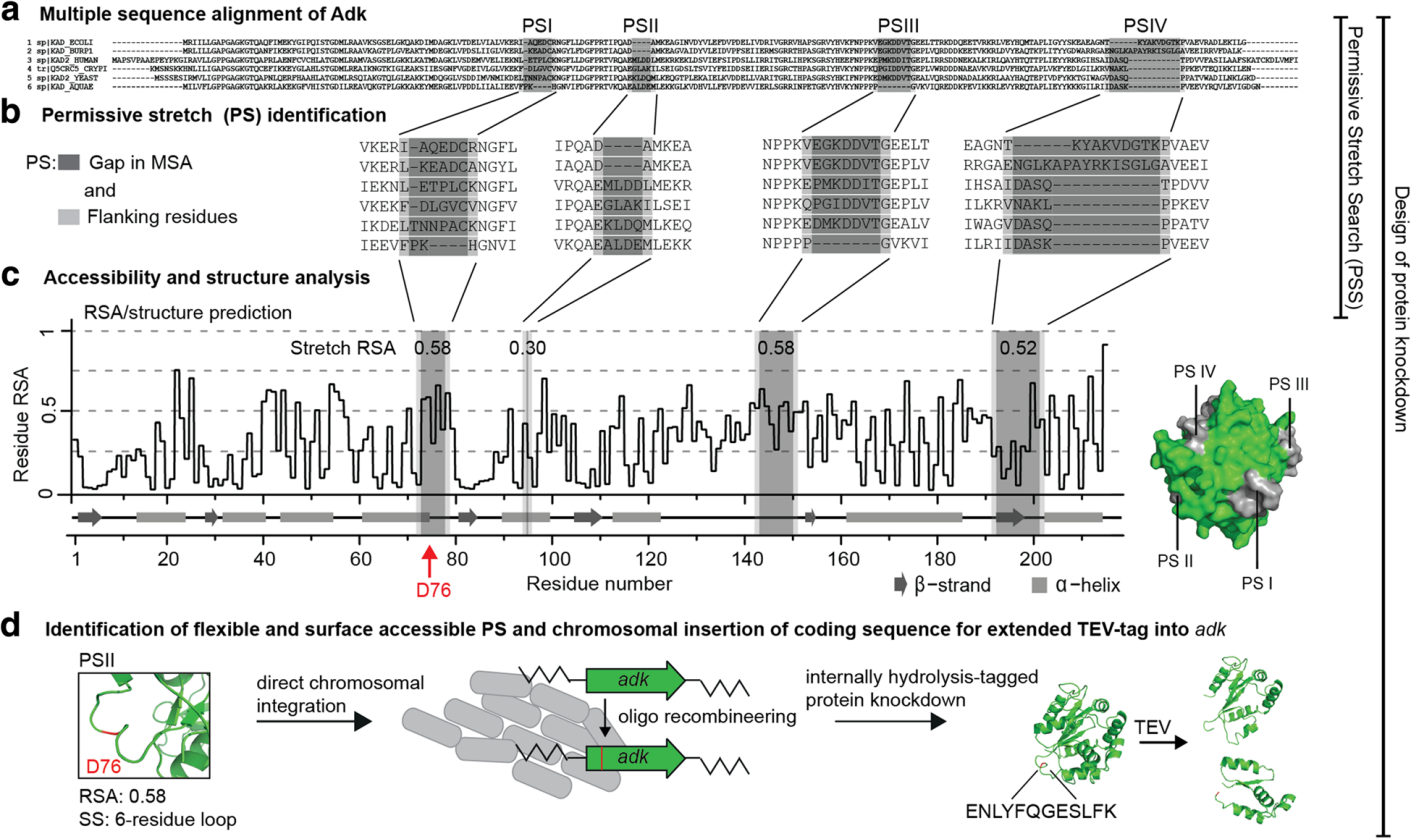
Established workflow for identifying permissive stretches (PSs) in proteins and design of protein knockdowns. The established workflow is exemplified with adenylate kinase (*Adk*) and requires primary structure information alone. **a** Gaps in a multiple sequence alignment (*MSA*) of several (>5) homologous proteins indicate stretches in a protein likely permissive to insertion of additional amino acid residues. **b** The span of a PS is defined as the gap in the alignment plus its flanking residues. The four identified PSs within Adk are indicated with Roman numerals. **c** The design of protein knockdowns requires the insertion of a Tobacco etch virus protease recognition site (TEV-tag) into a flexible, surface accessible PS. Relative surface accessibility (*RSA*) and structural context of a PS can be predicted based on primary structure information. RSA values for each PS within Adk are indicated and were calculated by computing the geometric means of the RSA values of adjacent residue pairs within a given stretch and taking their maximum value. RSA values range from 0 (buried) to 1 (fully exposed). The average maximum geometric mean RSA of a random stretch was determined to be 0.30. For illustration, PSs were mapped onto the surface representation of the crystal structure of Adk (Protein Data Bank (PDB) 1AKE). **d** The information acquired above guides the identification of a potentially functional, surface exposed, and flexible PS for chromosomal TEV-tag insertion. PSII shows a high RSA, and secondary structure prediction indicates that it stretches across a 6-residue loop. PSIII shows the same RSA as PSII and it stretches across an 18-residue loop. But PSII was shown to be functionally relevant ([42] and Fig. 2) and therefore not chosen for TEV-tag insertion

Here, we present a general method for predicting permissive stretches (PSs) in proteins. We hypothesise that length-variable regions (regions containing indels) in homologous proteins tolerate insertions. Such regions can be inferred by searching for gaps in a multiple sequence alignment (MSA) of homologous proteins. A similar strategy was employed to identify a permissive site in the glycoprotein of vesicular stomatitis virus [23], the yeast Ser/Thr kinases TOR1 and TOR2 [16], and the zebrafish proteins Tcf21 and Tbx18 [24], but the generality of this approach remained unclear. This procedure only requires primary sequence information, which is available for presumably any protein of interest. We further combine this method — which we call *permissive stretch search (PSS)* — with secondary structure and surface accessibility measures to establish a workflow that allows us to select permissive sites, which are structurally flexible and located in surface accessible regions (Fig. 1).

We apply this workflow to engineer conditional protein knockdowns for tailoring cell-free production platforms, in which the catalysts for complex biological processes can be recruited from living cells but employed outside the cell. Cell-free biotechnology is a quick and cost-effective method that allows for facilitated supply of non-membrane permeable or toxic substrates, easy monitoring, manipulation, and access to the desired products [25]. Cell-free platforms have shifted into focus for in vitro protein synthesis, for production of fine chemicals or medications [25–28], or for implementation of paper-based biosensors [29].

To prevent laborious and expensive purification schemes, we only disrupt the cellular envelope, leaving a crude lysate as the source of the required catalysts [26]. However, yield-efficient operation is then compromised by the presence of a complex enzymatic background that interferes with the desired reaction by sequestering starting materials, intermediates, and/or co-factors [26].

Here we tackle this problem by employing conditional protein knockdowns: we label enzymes with an internal Tobacco etch virus protease recognition site (TEV-tag) at a PS such that it can be inactivated at the cell-free stage through hydrolysis by a selective orthogonal protease. This allows us to target essential proteins that cannot be genetically inactivated or proteins that cause a growth phenotype in the biomass production strain in case of genetic elimination.

As a proof of principle, we address the rapid degradation of the expensive and universal co-factor adenosine triphosphate (ATP) and its hydrolysis product adenosine diphosphate (ADP), a problem that constrains the productivity of most cell-free production efforts [26, 30]. To avoid the addition of stoichiometric amounts of ATP, these cell-free processes rely on ATP regeneration from ADP. Stabilising the availability of the expensive co-factor ATP as well as its hydrolysis product ADP could allow for a more cost-efficient operation of cell-free production systems in general, especially when combined with existing strategies for ATP generation from glucose and the usage of nucleoside monophosphates (NMPs) as a source for the generation of nucleoside triphosphates (NTPs), which are required for messenger RNA (mRNA) production [31].

## Results

### Identification of functional PSs within proteins using sequence information alone

We first tested our hypothesis that length-variable regions are permissive to tag insertion with two *Escherichia coli* proteins for which various sites permissive to five-residue insertions had been experimentally identified by transposon mutagenesis previously: triosephosphate isomerase (TpiA, personal communication with Victor de Lorenzo, Additional file 1: Table S1) and TEM1 β-lactamase (Bla) [32]. For both proteins, sequences of four to six functionally conserved homologs with sequence identities ranging between 23% and 52% were selected and aligned. Input sequences are summarised in Additional file 2: Table S2 and alignments are given in Additional file 3: Figure S1.

The imposed sequence identity range was defined empirically: sequences with high similarity had very few gaps, while sequences with low identity exhibited too much alignment error, and their homology cannot be reliably determined from sequence information alone [33]. In general, an identity range between ~30% and ~70% works best in our view (see Additional file 2: Table S2 for input sequences and identity ranges). Transposon-identified permissive sites for TpiA and Bla were mapped onto the corresponding alignments (Additional file 2: Table S2 and Additional file 3: Figure S1). Remarkably, almost all of the experimentally identified permissive sites within TpiA (13 out of 16) and all within Bla (5 out of 5) mapped either directly to one of the identified gaps (9 out of 13 for TpiA and 4 out of 5 for Bla) or scattered close (within +/– 5 residues) to an identified gap (4 out of 13 for TpiA and 1 out of 5 for Bla). Scattered permissive sites — sites that are shifted from the actual gap in the specific alignment — tended to be at least part of the same secondary structural element as the identified gap. For Bla, semi-permissive sites (arbitrarily defined as conferring ≤ 30% wild-type resistance to ampicillin by the authors of the study) and non-permissive sites (loss of function) had been characterised as well [32]. Only 4 out of 10 semi-permissive sites mapped to one of the identified gaps, but perhaps more importantly, none of the non-permissive sites mapped to a gap.

Overall, this first analysis suggested that the predicted gapped regions in the alignments were indeed tolerant to insertion of additional amino acids. However, the gap search resulted in the identification of a potentially flexible stretch within a protein rather than a precise site. We therefore call this approach *permissive stretch search* (PSS) rather than permissive site search.

### Experimental validation of PSS with three test proteins

To experimentally validate the PSS, we used three test proteins which are either essential or conditionally essential: adenylate kinase (Adk), glycerol-3-phosphate dehydrogenase (GpsA), and the previously discussed triosephosphate isomerase (TpiA). MSAs for all test proteins, the identified PSs, and their numbering can be found in Additional file 4: Figure S2. We selected TpiA as a test protein for PSS — despite the fact that transposon-identified permissive sites for this protein are known — to validate the permissiveness of PSs that had not been transposon-identified (PSIV, Additional file 4: Figure S2), to sample additional sites scattering around transposon-identified PSs (site T130 scattering around PSVI, Additional file 4: Figure S2), and also to explore permissiveness of transposon-identified sites towards longer insertions (site E55 within PSIII and site T153 within PSVI, Additional file 4: Figure S2).

For all proteins, we inserted a TEV-tag (ENLYFQ↓G), or derivatives thereof, and explored insertion positions lying directly within predicted PSs as well as insertion positions scattering closely around them — similar to the distribution of our reference (transposon-identified) permissive sites. Varying the exact sites around the identified PSs is reasonable, as the exact gap position in an MSA can vary depending on the choice of alignment algorithm and the gap open and extension penalties that were employed [34, 35]. We also explored small deletions or duplications of the original sequence to study the extent of permissiveness for specific insert designs. Table 1 summarises the identified PSs for each protein and the final sequences of tagged protein variants. Input sequences and MSAs for all three proteins can be found in Additional file 2: Table S2 and Additional file 4: Figure S2.

**Table 1:** Overview of internally tagged protein variants

	Stretch	Span	Insertion site	Original sequence^a^	Sequence after insertion^b^
Plasmid insertions					
Adk	PSI	I72-R78	D76	QED**CRNGF**LLD	1-QED**ENLYFQG**LLD
	PSII	D94-A95	A93	PQA**DA**MKE	PQA**ENLYFQG**MKE
			K97	AMKEAG	AMK**ENLYFQG**EAG
			A99	KEAGIN	1-KEA**ENLYFQGMKEA**GIN
					2-KEA**ENLYFQGDAMKEA**GIN
	PSIII	V142-G150	P140	NPP**KVEGKDDV**TGE	NPP**ENLYFQG**TGE
	PSIV	T191-P201	A186	KEA**EAG**NTK	KEA**ENLYFQG**NTK
GpsA	PSI	P55-V57	C49	DRC**NAAFLPDVP**FPD	DRC**ENLYFQG**FPD
			P60	PFPDTL	PFP**ENLYFQGVPFP**DTL
	PSII	P97-D102	M99	PLMRPD	PLM**PTTENLYFQGCLG**RPD
	PSIII	L128-Q131	I132	DQIPLA	DQI**PTTENLYFQGCLG**PLA
	PSV	D272-V273	Q269	LGQGMD	LGQ**PTTENLYFQGGTV**GMD
TpiA	PSIII	E53-I59	E55	EAEGSH	1-EAE**GGSGENLYFQ** ***G*** **SGGS**GSH
					2-EAE**GCLGESENLYFQGDERKNK**GSH
					3-EAE**GCLPTTENLYFQSGTVKNK**GSH
	PSIV	D67-N69	N69	DLNLSG	DLN**ENLYFQG**LSG
	PSVI	E133-A156	T130	GET**EAENEAG**KTE	1-GET**ENLYFQGGSG**KTE
					2-GET**GGSENLYFQGGSG**KTE
			T153	LKTQGA	LKT**DYDIPTTENLYFQSGTVDAGAD**QGA
Chromosomal insertions					
Adk	PSI	I72-R78	D76	QEDCRN	3-QED**ENLYFQGESLFK**CRN
TpiA	PSIV	D67-N69	L70	LNLSGA	LNL**PPKNENLYFQGESLFKGP**SGA
AtpA	PSIII	H123-F126	H123	LDHDGE	LDH**ENLYFQG**DGE
AtpD	PSIII	E101-E105	E101	KGEIGE	KGE**ENLYFQG**IGE
Extended TEV-tag					
Adk	PSI	I72-R78	D76	QEDCRN	2-QED**ENLYFQG**CRN
					4-QED**ENLYFQGESLFKGG**CRN
GpsA	PSI	P55-V57	D56	LPDVPS	1-LPD **ENLYFQG** VPS
					2-LPD **PPKNENLYFQGESLFKGP**VPS

^a^Residues deleted during the insertion process are shown in boldface. If no residues were deleted, a 6-residue stretch of the protein sequence is shown, and the insert was placed in the middle

^b^Minimal TEV-tag and extended derivatives used for insertion are shown in boldface

Functionality of all protein variants was assessed in vivo by measuring the ability of a tagged protein variant to sustain wild-type-like growth rates on different carbon sources, a strategy frequently employed to examine functionality of protein variants [14, 36]. Adk is an essential protein required for the biosynthesis of purine ribonucleotides [37] and plays a key role in controlling the rate of cell growth by tuning the availabilities of nucleotide species via inter-conversion [38].

GpsA is also an essential protein, catalysing the first step in the biosynthesis of phospholipids starting from the glycolytic intermediate dihydroxyacetone phosphate (DHAP) [39]. As a central enzymatic activity in glycolysis and gluconeogenesis [40], TpiA is conditionally essential: it is non-essential for growth on rich media (Lysogeny broth (LB) medium), but essential if glucose or glycerol are the only carbon sources (M9 medium). Therefore, differences in the specific growth rates on a glycolytic carbon source (glucose) and a gluconeogenic carbon source (glycerol) should identify impairment of catalytic activity for the various Adk, GpsA, and TpiA variants once the wild-type gene on the chromosome is inactivated.

Protein variants were expressed from their natural promoters on low copy plasmids in strains lacking the corresponding wild-type gene in their chromosome. A wild-type version of each protein expressed from the same genetic context was used as a reference for growth rate comparisons. Note that due to the essential nature of *adk* and *gpsA*, the final strains had to be constructed by genetic replacement rather than standard transformation [41]. As *gpsA* is encoded within an operon, we included the upstream gene *secB* on the test plasmid to maintain its genetic context and tested TEV-tag carrying variants in strain *secB gpsA*::*kan*. Adk variants were tested in strain *adk*::*kan*, and TpiA variants were tested in the previously constructed double knockout strain *amn::FRT tpiA::FRT* [26].

**Fig. 2:**
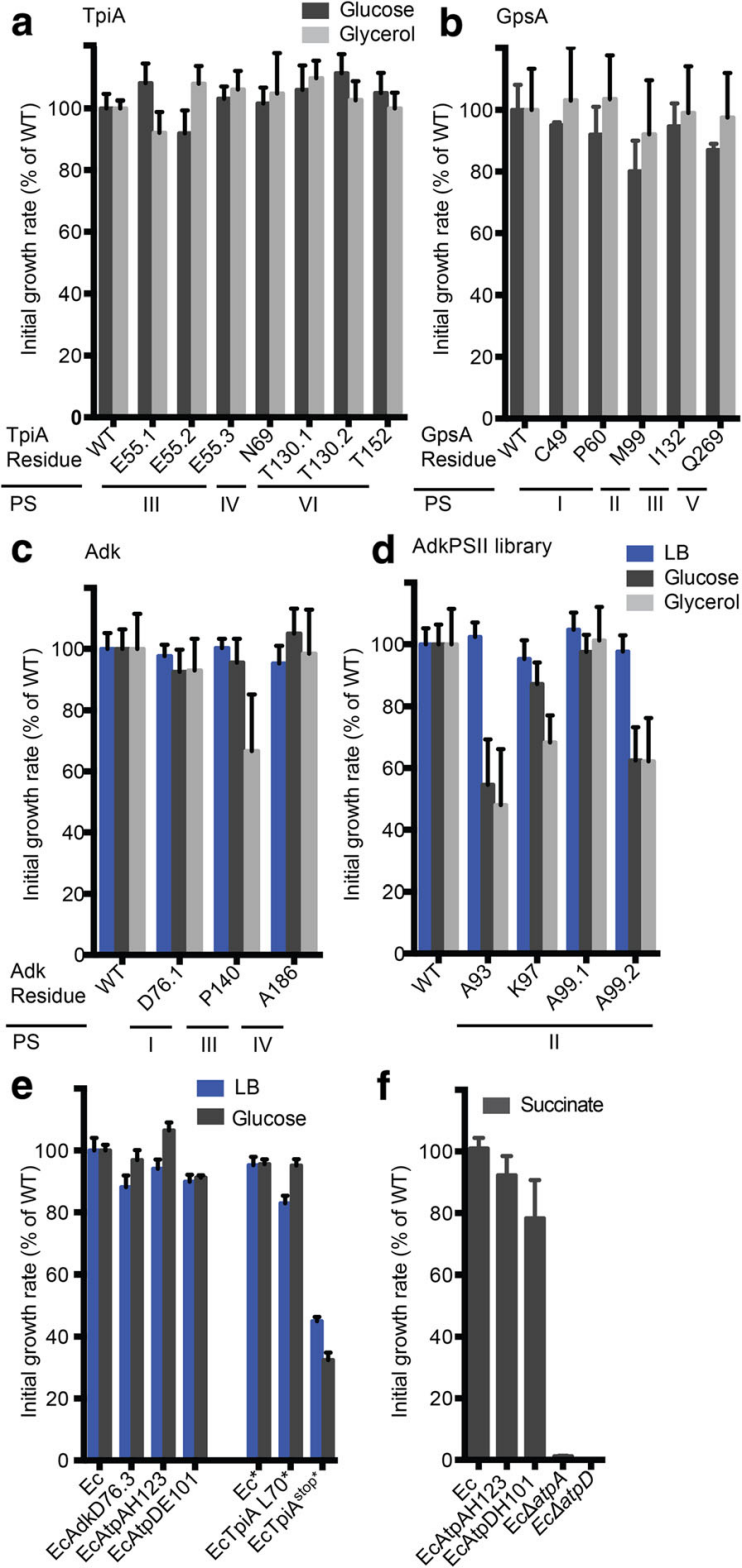
Functionality of TEV-tagged protein variants in vivo. *Upper and middle panels* (**a**-**d**): functionality of plasmid encoded TEV-tagged protein variants in vivo. Variants were expressed from their natural promoter on low copy plasmids. **a** TpiA, **b** GpsA, **c** Adk, **d** Adk variants isolated from an insertion library around PSII. Insertion positions and corresponding permissive stretches (*PSs*) are given for each variant. Functionality was evaluated as the ability of a certain variant to support growth of the corresponding knockout strain on different carbon sources at 37 °C. Experiments were done in biological duplicates ± SD. *Lower panel* (**e** and **f**): functionality of chromosomally encoded TEV-tagged proteins variants in vivo*.* **e** Indicated strains carrying a TEV-tag on the chromosome were grown in LB medium or M9 glucose with casamino acids at 32 °C, and growth rates were compared to the appropriate parent strain (*Ec* or Ec*) which was used for chromosomal integration; in case of TpiAL70 the strain has an additional STOP codon in *amn* (Ec*) resulting in a translational knockout. **f** Growth rates on M9 succinate of strains carrying a TEV-tag in the α- (AtpA) and β- (AtpD) subunits of ATP synthase. A functional ATP synthase is essential for growth on the non-fermentable carbon source succinate. Strains having AtpA or AtpD replaced by a kanamycin cassette fail to grow on succinate. Experiments were done in triplicate ± SD

For TpiA and GpsA, all of the tagged protein variants sustained wild-type-like growth rates on minimal media with glucose and glycerol (Fig. 2a and b). For Adk, TEV-tag insertions into two of four PSs (PSI and PSIV, see Additional file 4: Figure S2 for numbering) resulted in protein variants that could sustain wild-type-like growth on glucose and glycerol (Fig. 2c). Insertion into PSIII resulted in a variant with wild-type-like growth on complex LB medium and on minimal M9 medium with glucose but showed a growth defect on minimal medium with glycerol (66 ± 18% of wild-type-specific growth rate). As the literature indicated that PSIII is located in a functionally relevant loop [42], we excluded PSIII from further analysis. The TEV-tag insertion into PSII (specifically after residue A93) resulted in a variant that sustained wild-type-like growth on complex LB medium but caused a growth defect on M9 minimal media with glucose and glycerol (54 ± 14% and 47 ± 18%; mean ± standard deviation (SD) of wild-type-specific growth rate, respectively). This indicated that insertions at site A93 were not fully permissive, despite the lack of reports of the PSII region’s functional relevance. To explore if changing the exact insertion position within PSII could restore wild-type-like growth, we created a small insertion library by polymerase chain reaction (PCR) (Additional file 5: Figure S3). After screening several library members, we identified one variant (tagged after residue A99, with a four-residue duplication of the original sequence) sustaining wild-type-like growth on all carbon sources. Other screened insertion sites (after residue K97) and insertion designs (after residue A99, with a six-residue duplication of the original sequence) resulted in proteins with compromised function (Fig. 2d).

In summary, we examined a total of 11 PSs and 19 insert designs within three proteins and showed that 15 out of 19 designs resulted in functional protein variants. This included one permissive site within TpiA (N69), which had not been discovered by transposon mutagenesis. This suggested that PSS allows for a substantial reduction in effort from screening/selecting from a transposon library of variants to the functional evaluation of a few variants.

### Identified stretches are sufficiently permissive for chromosomal protein tagging

**Fig. 3:**
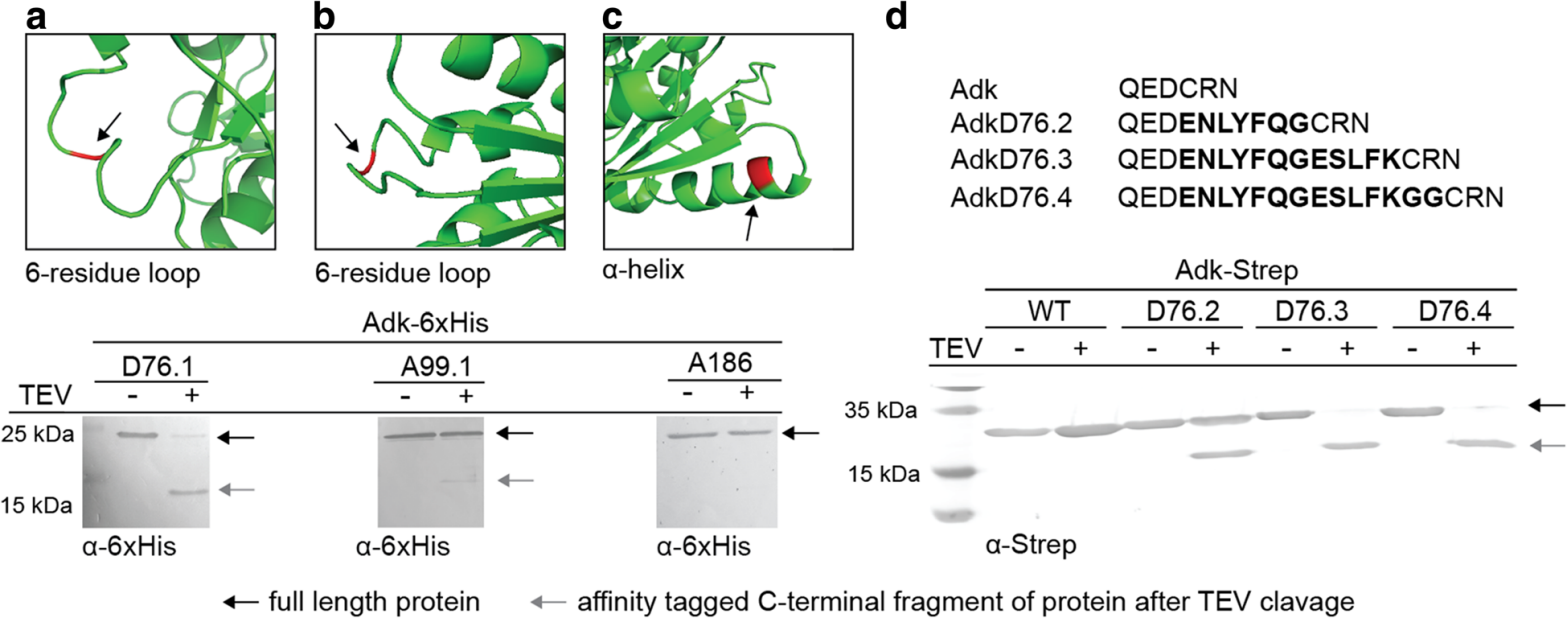
Cleavability of internally TEV-tagged protein variants. Cleavability of TEV-tagged Adk-6xHis variants was examined by incubating crude lysates derived from strains expressing the indicated Adk variant from a low copy vector under control of the natural Adk promoter in the presence or absence of TEV protease. Samples were separated by SDS PAGE and blotted. Cleavage products were detected with a 6xHis-tag-specific antibody. **a** AdkD76.1, **b** AdkA99, **c** AdkA186. The predicted secondary structure context of each residue is indicated in *red*. The loop length for variants AdkD76 and A99 was determined based on secondary structure prediction. **d** Cleavage of flanked TEV-tag variants inserted after residue D76. Purified AdkD76-Strep variants with differing TEV-tag flanking region lengths were incubated in the presence or absence of TEV protease and cleavage products were detected with a Strep-tag-specific antibody

Having confirmed that the protein variants are functional in principle, we proceeded to explore the effect of tag insertion when the encoding genes are present only in monocopy, which would correspond to our ultimate objective: designing and implementing internally tagged proteins on the chromosome of *E. coli* with only minimal testing and re-engineering. We chromosomally inserted TEV-tags into two of our test proteins, Adk and TpiA, using co-selection multiplex automated genome engineering (CoS-MAGE) [43]. We chose to integrate the two cleavable variants AdkD76.3 and TpiAL70, (Fig. 3d and Additional file 6: Figure S4). The constructed strains were designated EcAdkD76.3 and Ec*TpiAL70 (Table 1 and Additional file 7: Table S3). Wild-type-like growth rates on complex medium and minimal medium supplemented with glucose verified full functionality of both variants in vivo (Fig. 2e).

After confirming the wild-type-like function of strains that contain a chromosomal gene for a TEV-tagged protein variant designed by the PSS, we tested the procedure on the α- and β-subunits of the ATP synthase (AtpA and AtpD). ATP synthase is a complex multi-subunit molecular machine and should therefore be a very stringent target for verifying the PSS approach. We had also identified ATP synthase as a major source for unspecific ATP depletion in cell-free extract (CFX) (Additional file 8: Figure S5). We predicted PSs for AtpA and AtpD and inserted the nucleotide sequence for TEV-tags into the chromosomal gene sequence for two identified PSs, specifically after residues H123 (AtpA) and E101 (AtpD) (Table 1 and Additional file 9: Figure S6). The corresponding strains EcAtpAH123 and EcAtpDE101 exhibited 92 ± 6% and 72 ± 12% of wild-type growth rate on the non-fermentable carbon source succinate, indicating the assembly of a functional ATP synthase [44] (Fig. 2f). The strains further exhibited wild-type-like growth on complex medium and minimal medium supplemented with glucose (Fig. 2e).

These results suggested that the PSS could be used for direct chromosomal engineering, as tagged proteins were functional when expressed from a monocopy gene and targets could be chromosomally tagged with minimal testing effort.

### Ensuring accessibility of the inserted peptide tag: incorporating surface accessibility and secondary structure context shows that PSS-identified PSs are biased towards being surface accessible

Besides functionality, a further requirement for the usefulness of a permissive site is its accessibility to interaction partners, such as proteases, labelling enzymes, or antibodies. Depending on the partner, PS accessibility may not only be a function of simple surface accessibility but may also be dependent on secondary structure context, e.g. if the partner requires its recognition site to be located in a surface accessible unstructured loop. As we aimed to keep the entire workflow based on primary structure information, we used the freely available NetSurfP structure prediction tool [45] to assess relative surface accessibilities (RSAs) and secondary structures of each stretch.

We first verified that predicted results for RSA and secondary structure of a given protein correlated well with the structural data which were available for five of our test proteins, AtpA, AtpD, TpiA, Bla, and Adk (correlations ranging from 0.69 to 0.8 between sequence- and structure-based predicted RSA and secondary structure; Additional file 10: Table S4). Given this result and the good accuracy reported for NetSurfP’s predictions [45], we considered this tool sufficiently accurate for evaluating protein structure for PS selection in the absence of crystal structures.

Interestingly, when predicting the RSA for the PSs in our test proteins supported by manually mapping the positions of PSs onto available crystal structures, we realised that almost all of them were at least partly surface accessible (Fig. 1, Additional file 10: Table S4, and Additional file 11: Figure S7). Encouraged by this result, we were interested to evaluate if PSS-predicted stretches tended to be surface accessible in general. This would significantly support our goal to identify surface accessible PSs in a variety of proteins to eventually engineer protein knockdowns in a proteome-wide approach.

We therefore automated the PSS and predicted PSs across the functionally annotated part of the *E. coli* K-12 proteome. The most accessible insertion site (as defined in the Methods section) in predicted stretches displayed significantly higher RSA (0.389, 95% confidence interval (CI) = [0.0661, 0.712]) when compared to the most accessible insertion site in randomised stretches with the same length distribution (0.298, 95% CI = [0.0224, 0.574]) (see Methods). To evaluate the significance of this difference, we generated 1000 bootstrap samples of PS position shuffles to determine how these differences are distributed. We observed very little variability among the samples, with a mean average surface accessibility of 0.298, 95% CI = [0.297, 0.300]. This indicates that the higher average RSA of observed insertion sites is indeed statistically significant (*p* < 10^−3^, Additional file 12: Figure S8). In the sites located in our test proteins, we also found this significant enrichment with respect to RSA in PSs versus randomly chosen PSs: only 6 of 34 PSs are below, 14 of 34 PSs are within one SD, and 14 of 34 PSs within two SD above the mean RSA of a randomly placed site (Additional file 10: Table S4 and Additional file 11: Figure S7). These results supported that PSs identified by PSS exhibited higher than average RSAs, making it likely that using PSS in order to identify solvent exposed PSs could be generalisable to the proteome level.

### Design of conditional protein knockdowns: testing and improving cleavability of the minimal TEV-tag by adding flanking residues

Our specific interest in chromosomal protein tagging is the engineering of conditional protein knockdowns to enable easy elimination of undesired catalytic activities from a cell-free platform. This requires efficient hydrolysis of the primary peptide backbone by TEV protease and loss of enzymatic activity after cleavage.

When examining the cleavability of all functional Adk TEV-tagged variants by western blotting, we realised that the inserted minimal TEV-tag (i.e. the canonical and widely used sequence ENLYFQG) was generally poorly hydrolysed, and cleavage efficiency seemed to be dependent on specific sequence and secondary structure context. Specifically, the TEV-tag was partly hydrolysed (to different extents) when placed into a loop, as seen for AdkD76.1 and AdkA99.1 (Fig. 3a and b), but not at all when placed into an α-helix, as seen for AdkA186 (Fig. 3c). This finding was confirmed when examining the cleavability of all TEV-tagged variants of TpiA and GpsA (Additional file 13: Figure S9). Therefore, despite ensuring at least theoretically good access to tags, TEV hydrolysis seemed to require the consideration of additional criteria, and we developed a “flanked TEV-tag” that could be efficiently hydrolysed even when placed into a presumably structurally more rigid internal position.

Our flanked TEV-tag extends the minimal cleavage site by residues derived from one of the variable hydrolysis sites in the natural TEV polyprotein (UniProtKB: P04517). We tested variants of different length for improved cleavage and found that a TEV-tag minimal sequence extended by five residues at its C-terminus (ENLYFQ↓G ESLFK) substantially enhanced the cleavage efficiency of variant AdkD76.3 (Fig. 3d). The same strategy was successfully employed to engineer cleavable variants of GpsA and TpiA (Additional file 6: Figure S4).

### Stabilising the nucleotide pool in a cell-free platform using conditional protein knockdowns

As the first step, it was necessary to identify the major ADP and ATP sinks present in CFX. A database search for potentially abundant ADP consumers with no specific additional substrate or co-factor requirements yielded Adk as a strong candidate. As for ATP consumers, a database search for ATPases resulted in a set of potential candidates with uncertainty about abundance and activity under cell-free platform operation conditions. Therefore, ATP sinks were identified in a reverse approach by separating cell-free extracts on native PAGE, followed by activity detection and mass-spectrometric identification of corresponding proteins (Additional file 8: Figure S5). One identified major ATP sink was the soluble F_1_ portion of the membrane-spanning ATP synthase. Although membranes and membrane-bound proteins are removed during CFX preparation, the soluble F_1_ portion of ATP synthase is known to separate from the membrane-associated F_0_ part and remains present in CFX preparations. Without coupling to the proton-motive force, F_1_ hydrolyses ATP unspecifically [46, 47]. We therefore investigated tagging of Adk and two of the subunits of ATP synthase as possible measures for stabilizing nucleotides in CFX.

**Fig. 4:**
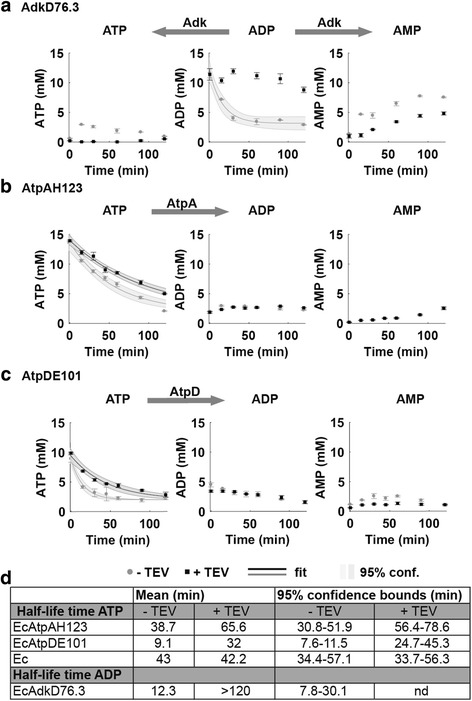
Stabilisation of nucleotide pool in CFX using conditional protein knockouts. Time course of nucleotide inter-conversion in CFX with or without pre-treatment by TEV protease **a** Adk: ADP was added to a CFX prepared from strain EcAdk76.3. **b** AtpA and **c** AtpD: ATP was added to a CFX prepared from strains EcAtpA and EcAtpD. Nucleotide concentrations were quantified at indicated time points by HPLC in triplicate ± SD. 95% confidence intervals indicated the accuracy of the fits. **d** Specific half-life times (min) with 95% confidence bounds (min) of ATP or ADP before and after knocking out enzymatic activity by TEV protease cleavage

We first tested for ADP stabilisation using the cleavable Adk variant (AdkD76.3; see above). We prepared a CFX from strain EcAdkD76.3 and used high-performance liquid chromatography (HPLC) to determine the stability of externally added ADP (and its metabolites) with or without pre-treatment by TEV protease (Fig. 4a). Fitting the data to an exponential decay model revealed that the half-life of ADP in CFX obtained from strain EcAdkD76.3 increased from ~10 min to greater than 2 h when Adk was inactivated by proteolysis (Fig. 4d). The interruption of inter-conversion of ADP to ATP and adenosine monophosphate (AMP) after proteolysis was verified by monitoring ATP and AMP concentrations (Fig. 4a).

Subsequently, to test for ATP stabilisation, we assessed the stability of ATP in a CFX prepared from strains EcAtpAH123 and EcAtpDE101 with or without pre-treatment by TEV protease. In the presence of TEV protease, the half-life of ATP increased twofold and threefold for inactivated AtpA or AtpD, respectively (Fig. 4b–d). ATP half-life in a CFX prepared from the wild-type strain Ec was not affected by protease treatment (Additional file 14: Figure S10). We verified by western blotting that AtpAH123 and AtpDE101 were indeed cleaved by TEV protease (Additional file 15: Figure S11).

## Discussion

We developed and tested an approach for predicting PSs in proteins to enable the rational design of internally tagged proteins based on primary structure information alone. We validated our approach by harnessing existing literature data on permissive sites in bacterial proteins as well as by experimentally verifying predicted PSs in various *E. coli* proteins.

Our approach can minimise the number of design, test, and re-engineering cycles, enabling efficient internal protein tagging directly into the genome. We exemplify this by functionally tagging AtpA and AtpD directly on the *E. coli* chromosome in a *single* engineering cycle. Both our literature-derived and our own experimental data suggest that PSs are permissive to insertions of various lengths at different positions within a stretch. Additionally, in a proteome-wide analysis, we show that identified PSs are enriched in surface accessible regions, making them suitable candidates for tag interaction.

However, during the engineering of conditional protein knockdowns, we observed that surface accessibility is not the only requirement for efficient hydrolysis of a TEV-tag by the TEV protease. Inefficient hydrolysis of internal TEV-tags has already compromised other engineering efforts [17], and therefore, we wanted to exclusively insert TEV-tags that could be efficiently hydrolysed. Hydrolysis seems strongly dependent on the structural context of the chosen stretch. In fact, cleavage of our tested proteins was only achieved when the TEV-tag was inserted into a structurally flexible loop region and could be sufficiently improved by extending the minimal TEV-tag by additional residues derived from one of the TEV polyprotein cleavage sites. Proteases frequently recognise their substrates in an extended β-strand conformation [48], and we assume that adding flanking residues to the TEV-tag gives the substrate more flexibility to adopt the correct conformation.

To demonstrate the applicability of our approach, we designed conditional protein knockdowns to engineer a cell-free platform with enhanced ADP and ATP stability after TEV cleavage. The single-protein knockdown of Adk could almost completely halt drainage of ADP over a time span relevant for cell-free protein production [49] or biotransformations [26]. In addition, ATP half-life could be stabilised two- to threefold by employing single-protein knockdowns. These results were very encouraging given that our activity mapping showed ATP degradation in CFXs to be complex and several further potential sinks were identified, suggesting that proteolytic elimination of additional enzymes could further enhance stability. Still, it remains to be tested if the herein achieved enhancement of ATP stability in CFX leads to improved performance of a cell-free system, e.g. for small molecule or protein production.

Our protein knockdowns can be chromosomally implemented by well-established and cheap oligo-recombineering, and inactivation depends on a single component (TEV protease), which does not require co-factors. Other imaginable knockdown strategies like induced mRNA decay [50] or terminal degradation tags [51] would require extensive recoding of genes (in case of mRNA decay) for implementation and would rely on cellular machinery and co-factors (ATP) to achieve a protein’s knockdown.

## Conclusions

Based on our analysis of existing data, experimental verifications, and proteome-wide predictions, we suggest that this method is of general utility. Correspondingly, we have developed design guidelines consisting of four steps (identification of PSs by searching for gaps in functionally conserved homologous proteins; determination of PS accessibility; determination of loop flexibility; genomic integration) for successfully engineering internally tagged proteins and inserting them into the chromosome (see Design guidelines). Although established in *E. coli*, we believe that the basic concept of PSS is widely applicable to proteins from different species across kingdoms. We are aware that, in higher organisms, post-translational modifications, splicing, or protein-protein interactions play a more important role and will need to be considered. PSS leaps beyond state-of-the-art methods for permissive site identification and allows for the rapid and parallel design and implementation of engineered proteins. This is essential for systematic protein engineering efforts like the herein presented cell-free platform engineering, where we envision protein knockdown multiplexing on a whole-proteome scale. We also emphasise the simplicity of our approach: harnessing the vast repository of protein sequences contained in sequence databases as the sole input results in straightforward design of internally tagged proteins.

### Design guidelines

Based on our analysis, we provide general design guidelines for successful engineering of internally tagged proteins and their chromosomal insertion.

#### Step 1: Identification. Search for gaps in functionally conserved homologous proteins

For MSA construction and gap identification, we recommend aligning at least four to six functionally conserved protein sequences from different species. The percent identity of the chosen sequences should be sufficiently high with respect to the selected search algorithm to prevent misidentification of homologs — which could potentially introduce incorrect gaps into the MSA [34] — while ensuring that the chosen sequences are dissimilar enough to reduce sampling bias. We observed that permissive sites scattered around identified PSs, which indicates that there is some freedom in insert site selection around PSs. To avoid false positives, we recommend considering, when available, literature information on important functional features of a protein or a protein family, to avoid disrupting components known to be important for function.

#### Step 2: Accessibility. Find an accessible PS

Although we found predicted PSs to be enriched for surface accessibility, we recommend verifying the accessibility of the location of a stretch. Predicted surface accessibility [44] is a good proxy if structural data are unavailable. For partially buried stretches, a user can simply choose an exposed position within the stretch.

#### Step 3: Flexibility. Find a stretch within a flexible loop region

Some applications require more stringent criteria than surface accessibility. As shown herein for the TEV-tag, but also known for other tags [3], the structural context of a PS might be relevant to the function of a peptide tag. Thus, we suggest evaluating the secondary structure context by examining available three-dimensional structural data or using secondary structure prediction.

#### Step 4: Genomic integration

We demonstrate that inserts can be integrated into the chromosome, allowing for genomic tagging of proteins. Although we specifically used MAGE for genomic insertion of TEV-tags, we emphasise that any precision genome-editing tool can be used, since the design and implementation of tagged proteins is de-coupled. Therefore, we recommend carefully choosing the most suitable genome-editing tool for the relevant host.

## Methods

### Chemicals and enzymes

Restriction enzymes, T4 ligase, and the Gibson assembly kit were obtained from New England Biolabs (Ipswich, MA, USA) and used according to the manufacturer’s instructions. Chemicals were purchased in the highest purity available from Sigma-Aldrich (St. Louis, MO, USA), Fluka (Buchs, Switzerland), or Roth (Lauterbourg, France).

Trypton, yeast extract, Bacto™ casamino acids, Low salt Difco™ LB Base, Miller (LB Miller), and Difco™ MacConkey agar base were obtained from BD Bioscience (Basel, Switzerland). Low salt LB-Miller medium was used to grow cells for the MAGE experiments, and chloramphenicol at 20 μg mL^−1^, kanamycin at 50 μg mL^−1^, or carbenicillin at 50 μg mL^−1^ was supplied for antibiotic selection. Isopropyl β-D-1-thiogalactopyranoside (IPTG) was added to 0.1 mM and 5-bromo-4-chloro-indolyl-β-D-galactopyranoside (X-gal) to 40 μg mL^−1^ to LB agar plates for blue/white selection for LacZ functionality. MalK functionality was tested on MacConkey agar (40 g L^−1^) supplemented with 10 g L^−1^ d-(+)-maltose monohydrate. Complex LB medium contained 10 g L^−1^ trypton, 5 g L^−1^ yeast extract, and 10 g L^−1^ NaCl. M9 minimal medium contained 1× M9 salts [52] and was supplemented with 10 mg L^−1^ thiamine, 2 mg L^−1^ biotin, and the carbon source as mentioned in the text.

For affinity purification of proteins, Strep-Tactin® purification resins (iba, Göttingen, Germany) or Ni-NTA agarose (Thermo Fisher, Reinach, Switzerland) was used using the recommended buffers of the corresponding supplier.

Desalted oligonucleotides and Sanger sequencing services were obtained from Microsynth (Balgach, Switzerland) and Sigma-Aldrich (St. Louis, MO, USA). MAGE oligos were purchased with 4-phosphorothiolated bases at the 5’ end.

### Strains, plasmids, and primers

For lists of strains, plasmids, and primers, see Additional file 10: Table S4, Additional file 16: Table S5, and Additional file 17: Table S6.

### Growth rate determination

Determination of initial growth rates of strains carrying plasmids with TEV-tagged protein variants was performed in 5 mL LB or M9 minimal medium supplemented with 0.5% glucose or 1% glycerol and 0.2% casamino acids at 37 °C. Samples were taken every 60 min, transferred to a 96-well plate, and OD_600_ was determined in a Viktor^3^ 96-well reader from Perkin Elmer (Schwerzenbach, Switzerland). Chromosomally tagged variants were tested at 32 °C (all EcNR1-based strains contain a heat inducible lambda red system and therefore cannot be cultivated at 37 °C) in one of the following media (as specified in the main text): LB medium, M9 minimal medium supplemented with either 20 mM succinate and 0.05% yeast extract or 0.5% glucose and 0.2% casamino acids. Growth rates were measured optically with a BioLector in a 48-well FlowerPlate from m2p-labs GmbH (Baesweiler, Germany).

### TEV protease and protease cleavage in CFX

For cleavage, we used two different TEV protease versions. For initial small-scale cleavability determination, we used an N-terminally Strep-tagged TEV_opt_ version that was affinity purified using Strep-tactin resin as indicated by the manufacturer (iba, Göttingen, Germany). TEV_opt_ was codon optimised for *E. coli*, and carries the following substitutions, which were reported previously to enhance solubility or functionality [51, 53]: N68D, S219V, last six residues deleted. An aliquot of 50 μg of TEV_opt_ was used per milligram of CFX.

For optimisation of cleavage and proteomic switching, we used a TEV protease variant which is expressed as a cleavable C-terminal fusion with maltose binding protein (MBP) and is equipped with a 6xHis-tag at the N-terminus [54]. It was purified by Ni^2+^-NTA affinity purification followed by dialysis against TEV buffer (10 mM sodium phosphate buffer pH 7.5 with 1 mM dithiothreitol (DTT) and 1 mM ethylenediaminetetraacetic acid (EDTA)). An aliquot of 5 μg of TEV per milligram of CFX was used for proteomic switching experiments and 1:5 (wt/wt) TEV:protein for purified protein samples in TEV buffer. Typically, the first one third of the total amount of TEV protease was added followed by incubation at 30 °C for 2 to 3 h, after which time another third of the TEV was added, again followed by incubation for 3 to 4 h at 30 °C. Afterwards the mix was centrifuged at 20,817 × g and 4 °C for 30 min, and the last third of TEV was added to the supernatant and incubated overnight at 4 °C.

### Cleavage analysis of purified Adk variants

Adk variants with a Strep-tag were cloned by Gibson assembly into vector pKTS [55]. For overexpression, *E. coli* BL21 cells with the corresponding plasmid were grown in LB medium at 37 °C until an OD_600_ of around 0.6 and induced by 100 ng mL^−1^ anhydrotetracycline (aTc). Six hours after induction, the cells were harvested and the Adk protein purified on a Strep-Tactin spin column. The purified Adk was TEV treated as mentioned above.

### Protein detection

Protein cleavage was analysed by western blot; the cleaved proteins were separated on an SDS gel of appropriate concentration and blotted on a nitrocellulose membrane of pore size 0.4 μm (GE Healthcare, time and voltage depending on protein size). Proteins were detected using a monoclonal mouse 6xHis-tag antibody (Qiagen, Hilden, Germany) for Adk, GpsA, and AtpD variants, a mouse monoclonal Strep-tag antibody (Qiagen, Hilden, Germany) for AtpA variants, or a rabbit polyclonal E-tag antibody (Abcam, Cambridge, UK) for TpiA variants (LabForce AG, Nunningen, Switzerland). A goat anti-mouse IgG-alkaline phosphatase conjugate (Sigma-Aldrich, Buchs, Switzerland) or a goat anti-rabbit IgG-alkaline phosphatase conjugate (Sigma-Aldrich, Buchs, Switzerland) in combination with a chromogenic alkaline phosphatase reagent kit (Thermo Fisher, Reinach, Switzerland) was used for detection. Purified Adk proteins were probed by a mouse anti-Strep monoclonal antibody (2-1507-00; dilution 1:10,000; iba, Goettingen, Germany) followed by detection by IRDye® 800CW goat anti-mouse (925-32210; dilution 1:10,000; LI-COR, Bad Homburg, Germany).

### Chromosomal integration

The DNA sequences for inserted peptides were delivered to chromosomal genes by CoS-MAGE. General procedures were carried out as described earlier [43]. More specifically, the oligonucleotides were designed to insert the sequence for the 21-bp TEV-tag or the flanking regions at the gene site corresponding to the permissive site of the protein of interest (see Additional file 16: Table S6 for specific primers). The oligonucleotides targeted the lagging strand of the chromosome and were optimised to have a ∆G of > –12.5 kcal/mol (determined with Mfold [56]). Additionally, oligonucleotides for translational knockouts for *tpiA* and *amn* were designed with the online platform MODEST [57]. For CoS-MAGE, 3 mL of *E. coli* (Ec) were grown in LB medium at 32 °C until an OD_600_ of around 0.6 was obtained. Cells were then heat-shocked for 15 min at 42 °C to induce the lambda red genes. An aliquot of 2 mL of induced cells was made electrocompetent by washing three times with ice-cold water. Then, 2 μM of each TEV-tag oligo and 0.2 μM of the co-selection oligo were added to the cells and the cells were electroporated (1-mm gap cuvettes, 1.8 kV). Cells were recovered by the addition of 3 mL of fresh LB Miller medium for further MAGE cycles. After two cycles of MAGE, the cells were recovered overnight at 32 °C and spread on selective medium plates, the specific composition of which depended on the selection marker (LB agar plate with the selective antibiotic for *bla* (ampicillin) or *rpsL* (streptomycin); McConkey agar plate with maltose for *malK*). Clones were analysed by Colony PCR (Multiplex PCR Qiagen). For PCR primers see Additional file 16: Table S6. The PCR program was performed as follows. Step 1: 15 min at 95 °C; step 2: 30 s at 95 °C; step 3: 30 s at 50 °C; step 4: 60 s at 72 °C; repeat steps 2 to 4 30 times; step 5: 10 min at 72 °C and storage at 8 °C. The insertions were verified by Sanger sequencing of PCRs of the genomic regions.

### Preparation of CFX

Cultures of the appropriate strain were cultivated in LB medium and harvested at an OD_600_ of around 2.8 by centrifugation. Cell pellets were re-suspended 1:1 (cell wet weight to buffer volume) in 10 mM sodium phosphate buffer (pH 7.5 and disrupted by homogenisation with EmulsiFlex-C3 (Avestin Europe GmbH, Mannheim, Germany) at a pressure of 1500 bar. Cell debris was pelleted by centrifugation at 40,000 × g and 4 °C for 30 min, and the supernatant was used as a CFX or stored at –80 °C. The protein concentration in the CFX was determined by a standard Bradford assay [58]. Protein concentrations in CFX were around 15–20 mg mL^–1^ for the different samples (compared to bovine serum albumin as standard). Prior to use, the CFX was centrifuged again (21,130 × g and 4 °C for 30 min to remove denatured proteins)

### ATP and ADP stability assay

In order to determine the stability of ADP and ATP in CFX, 15 mM ATP or 15 mM ADP was incubated in 10 mg mL^–1^ CFX in 10 mM sodium phosphate buffer (pH 7.5) with additional 1 mM MgCl_2_, 10 mM KCl, and 1 mM DTT, and three samples were withdrawn at each sampling time (30 μL). Proteins were immediately precipitated by the addition of 30 μL ice-cold isopropanol and subsequent centrifugation at 21,130 × g and 4 °C for 30 min. Samples were 1:1 diluted with double-distilled water (ddH_2_O) and 2-μL aliquots were analysed by HPLC in an Agilent Series 1200 device equipped with an auto-injector, an Accucore aQ (2.6-μm particle diameter, 150 × 4.6 mm^2^ column dimensions, Thermo Fisher Scientific, Reinach, Switzerland) column, and a UV monitor set to 254 nm. An isocratic elution was performed with 50 mM potassium phosphate pH 6 at a flow rate of 0.7 mL min^–1^. Peaks for ATP, ADP, and AMP were identified and quantified by retention times and comparison with authentic standards.

In order to compute the half-life, the concentration data were fitted to an exponential decay model using the Levenberg-Marquardt non-linear least squares algorithm from the MATLAB R2016b (Mathworks, Natick, MA, USA) curve fitting toolbox. We used the decay model

$$ \frac{\left[ ATP\right]}{\mathrm{mM}}=2+b{e}^{t{c}^{-1}} $$

to fit the initial concentration (2 + *b*) mM and ln(2) ⋅ *c* for the ATP half-life (where *t*, *c*, and the half-life are in minutes, and *b* is unitless). The asymptote was set to 2 mM, as the ATP concentration did not drop below this level within 4 h in previous experiments (data not shown). Since no asymptote could be determined experimentally for ADP decay, we used the model

$$ \frac{\left[ ADP\right]}{\mathrm{mM}}=d+b{e}^{t{c}^{-1}} $$

instead and fit *d* as well, where (*d* + *b*) mM is the initial concentration. Finally, 95% parameter CIs were plotted for both models.

### Manual PSS

Relevant sequences were retrieved from the functionally annotated database Universal Protein Resource Knowledgebase (UniProtKB) [59] using domain enhanced lookup time accelerated BLAST (DELTA-BLAST) [60]. Alignments were performed using the online version of Clustal Omega with default parameters [61]. Input sequences and UniProtKB accession numbers for all MSAs are summarised in Additional file 2: Table S2.

The span of each PS was defined by the two flanking residues of the gap in the underlying alignment, and its surface accessibility assessed by the maximum geometric mean RSA of adjacent residue pairs within the PS.

### Correlation of crystal structures and predictions for secondary structure and RSA

Crystal structures were obtained in Protein Data Bank (PDB) format from the Research Collaboratory for Structural Bioinformatics (RCSB) Protein Data Bank [62]. Secondary structure and absolute surface areas (ASAs) for each residue were obtained from the DSSP database [63], while relative surface areas, a measure of RSA, were computed from ASAs using the Bio.PDB.DSSP Python module [64]. RSA and secondary structure predictions were performed using the NetSurfP tool [45]. Given predictions, the secondary structure context assigned to each residue was defined as the annotation (helix, strand, or coil) with the highest probability.

Correlation of predicted and crystal structure surface accessibilities was assessed by computing the distance correlation [65] between the per-residue RSA values. To assess the correlation of secondary structures, annotations were mapped onto the set of points

$$ SS=\left\{\left(\begin{array}{c}0\\ {}0\end{array}\right),\left(\begin{array}{c}1\\ {}0\end{array}\right),\left(\begin{array}{c}1/2\\ {}\sqrt{3}/2\end{array}\right)\right\} $$

to ensure that all annotation types are equidistant. Distance correlation was then calculated in this space.

### Automated proteome querying and homolog retrieval

As a sample set to test if PSs are enriched in surface accessible regions, 4434 proteins in the UniProtKB/Swiss-Prot database [59] were chosen from the *E. coli* K-12 proteome [66]. RSA and secondary structure were predicted for each residue using the NetSurfP tool [45] and, when available, obtained from crystal structures from the DSSP database [63]. Homologs were retrieved using the DELTA-BLAST tool [60], searching against the version of the UniProtKB/Swiss-Prot database from March 23, 2016 and the conserved domain database from May 27, 2015. With composition-based statistics disabled, all search hits with an E-value below 0.001 were retrieved. The hits were subsequently filtered to discard those whose high-scoring pairs (HSPs) covered less than 80% of the query length or had no inserts. Since the sensitivity of DELTA-BLAST drops significantly below a sequence identity of 30% [60], all hits below this cut-off were also discarded. Of the 4434 proteins tested, 4097 had at least one homolog in the Swiss-Prot database, and 2613 had at least one search hit satisfying the filtration criteria.

### Detecting PSs from HSPs

Given a query protein and its respective homologs, a PS in the query is defined as a gap in their MSA plus its immediate flanking residues. To avoid the expense of computing an MSA for each query protein for this analysis, indels in the HSPs for the query proteins were used to construct PSs. We justify this approximation by noting that the exact positions and lengths of stretches are dependent on choice of alignment algorithm and gap scoring model. We define a PS of a query protein precisely using its HSPs in the following manner.

Let *p* denote a query protein in the proteome *P* and let HSP(*p*, *i*) be its i^th^ HSP. We define the *gap set* of HSP(*p*, *i*) as g(*p*, *i*) = {[*s*, *e*]|indel in HSP(*p*, *i*) in (*s*, *e*)}, where *s* and *e* denote the left and right flanks of the indel, respectively. Using this, the set of PSs of *p*, PS(*p*), is defined as:

$$ PS(p)=\left\{\tilde{g}\left|\tilde{g}\ \mathrm{connected}\  \mathrm{component}\  \mathrm{of}\ \underset{i}{\cup }g\left(p,i\right)\right.\right\} $$

### Testing PSs for enrichment in accessible regions

After detecting PSs in the sample set of proteins, the stretches were tested for significant enrichment in surface accessible regions, i.e. ${H}_a:{E}_{{\left\{ PS\left({p}_i\right)\right\}}_i}\left[ RSA\right]>E\left[ RSA\right]$, by generating 1000 bootstrap samples of PSs site shuffles. Using the definition of the RSA of the i^th^ residue of the query, RSA(*i*), the RSA of a PS is defined as $RSA\left(\left[s,e\right]\right)= ma{x}_{i\in \left\{s,\dots, e-1\right\}}\sqrt{RSA(i) RSA\left(i+1\right)}$.

The RSAs of the sites preceding the N-terminus and proceeding C-terminus are defined to be the RSAs of the first and last residues, respectively.

To generate a sample, each stretch [*s*_*m*_, *e*_*m*_] was assigned to a random protein *p*_*k*_ with a probability proportional to *p*_*k*_'s length. Then for each protein *p*_*k*_, its assigned stretches were distributed uniform-randomly in the protein. This process was repeated to generate each bootstrap sample ${\widehat{PS}}_j(P)$. The statistic ${\mu}_{{\widehat{PS}}_j(P)}(RSA)$ was then computed for all bootstrap samples to generate the null distribution of mean RSAs. The test statistic *μ*_PS(*P*)_(RSA) was evaluated for significance against this distribution.

## Additional files

Additional file 1: Table S1. Mapping of known permissive sites within TpiA and Bla. A known permissive site (first column, given as residue number) within TpiA and Bla [32] was assigned to a predicted stretch (PS) if it mapped directly within a PS (see Additional file 3: Figure S1 for illustration) or if it was shifted by few residues but at least mapped to the same secondary structural element (“scattered” permissive sites). The number of residues by which a given “scattered” site is shifted is given in column 5. Note that, due to the experimental approach, which selected for only functional protein variants, there are no known non-permissive sites for TpiA. (DOCX 90 kb)

Additional file 2: Table S2. PSS input sequences and accession numbers. (XLSX 42 kb)

Additional file 3: Figure S1. Mapping of previously known permissive sites within TpiA and Bla onto the corresponding MSA. (a) E. coli triosephosphate isomerase (TpiA), (b) *E. coli* TEM1 β-lactamase [32]. Known permissive sites are summarised in Additional file 1: Table S1. Predicted permissive stretches (*PSs*) are highlighted in *grey. Dark blue arrows* functional permissive sites, *light blue arrows* semi-permissive sites (sequence insertion altered the function to some extent), *red arrows* non-permissive sites. Predicted secondary structure is given above the alignment. *Light grey boxes* depict α-helices, *dark grey arrows* depict β-strands, and the *grey line* depicts unstructured coils. Note that due to the experimental approach, which selected for only functional protein variants, there are no known non-permissive sites for TpiA. (PNG 429 kb)

Additional file 4: Figure S2. Identification of permissive stretches in Adk, GpsA, and TpiA. MSAs for Adk (a) GpsA (b), and TpiA (c). Identified permissive stretches are highlighted in *grey*. Residues which were chosen for TEV-tag insertion are given as *red numbers* above the alignment. The predicted secondary structure for both proteins is given above the alignments. *Light grey boxes* indicate α-helices, *dark grey* depict β-strands. (PNG 631 kb)

Additional file 5: Figure S3. Construction of Adk test library. A TEV-tag insertion library around PSII of Adk was generated by vector PCR using a pool of different primers. The primer design allowed for different insert designs such as simple insertion, replacements, and duplications. We sampled the region, spanning residues A93 to A99 for potential insertions. The identified gap in our alignment spans D94 and A95 of *E. coli* Adk. Sequences of four clones are given, which were selected for further analysis. (PNG 58 kb)

Additional file 6: Figure S4. Engineering cleavable TpiA and GpsA variants using an extended TEV-tag. (a) TpiAL70 is located in a 15-residue loop (Additional file Additional file 4: Figure S2c and Additional file Additional file 11: Figure S7c). An extended TEV-tag flanked by C- and N-terminal extensions derived from the TEV polyprotein was inserted after position L70. Hydrolysis of the variant TpiAL70 by TEV protease was tested as described for Additional file Additional file 13: Figure S9. Note that Additional file Additional file 13: Figure S9b verifies that the minimal TEV-tag inserted after residue N69 is not cleaved by TEV protease. TpiA was C-terminally 6xHis-tagged and detected with a 6xHis-specific antibody. (b) GpsAD56 is located in a 16-residue loop, as predicted by secondary structure prediction (Additional file Additional file 4: Figure S2b and Additional file Additional file 11: Figure S7h). The minimal TEV-tag as well as an extended TEV-tag flanked by C- and N-terminal extensions derived from the TEV polyprotein were inserted after position D57. Hydrolysis of the corresponding variants GpsAD57.1 and GpsAD57.2 by TEV protease was tested as described for Additional file Additional file 13: Figure S9. GpsA was C-terminally 6xHis-tagged and detected with a 6xHis-specific antibody. (PNG 83 kb)

Additional file 7: Table S3. Strains used in this study. (DOCX 20 kb)

Additional file 8: Figure S5. Systematic identification of ATP sinks in CFX. CFX was fractionated by step-wise (increments of 10% per step) ammonium sulphate precipitation. Fractions were separated by native PAGE. The gel was subsequently incubated in ATP and stained to detect liberated inorganic phosphate (*Pi*) by malachite green as described [67] (*lower panel*). In brief: The gel was dipped for 30 min in redox buffer (30 mM Tris/HCl, 80 mM KCl, 5 mM MgCl2 10 mM DTT), followed by 1 h incubation in 10 mL substrate buffer (30 mM Tris, 80 mM KCl, 5 mM MgCl2, 10 mM ATP) at 37 °C. We added 2 mL of a malachite green solution (1.2 mL 0.44 mg malachite green in 100 mL H2O/H2SO4 and 0.8 mL 7.5% ammonium molybdate) directly to the substrate buffer. Colour development was allowed to proceed for 1 h. Samples were split after fractionation and additionally with Coomassie Blue to visualise all present protein bands (*upper panel*). *Dark green spots* indicate the presence of ATPase activity. Spots were cut from the gel and proteins were identified by mass spectrometry. *White arrows spots* corresponding to GroEL, *red arrows spots* corresponding to the F1 part of ATP synthase *grey arrow* spot corresponding to DnaK; purified GroEL was used as a positive control (C). (PNG 344 kb)

Additional file 9: Figure S6. Identification of permissive stretches in the α- and β-subunits of Fo part of ATP synthase. MSAs were generated for AtpA (a) and AtpD (b). Permissive stretches are highlighted in *grey*. Insertion positions are given in *red*. The predicted secondary structure for both proteins is given above the alignments. *Light grey boxes* indicate α-helices, *dark grey arrows* indicate β-strands. (PNG 613 kb)

Additional file 10: Table S4. Structural features of predicted permissive stretches determined by structure prediction and crystal structures. Secondary structure (SS) and RSA were predicted from sequence using NetSurfP [45] and calculated from crystal structures using the DSSP algorithm [63]. A crystal structure for GpsA was not available. The RSA of a stretch is defined as the maximum RSA of its constituent sites. The correlations of RSA and SS between predictions and crystal structures at all residues were computed by distance correlation [65]. The crystal structure was not resolved in TpiA PSIV and AtpA PSI. See Methods for more precise definitions of these calculations. (DOCX 68 kb)

Additional file 11: Figure S7. PSS-identified permissive stretches are enriched to be surface accessible. Each plot summarizes the predicted surface accessibility and the predicted secondary structure of each residue for all test proteins (*upper panel within each plot*) as well as relative surface accessibility and secondary structure calculated from available crystal structures by the DSSP algorithm (*lower panel within each plot*). (a) Adk, (b) Bla, (c) TpiA, (f) AtpA, (g) AtpD, and (h) GpsA. PSs are highlighted in grey. The RSA of a site is defined as the geometric mean of the RSAs of its flanking residues, while the RSA of a predicted stretch is defined as the maximum site RSA in the stretch. RSAs of stretches derived from predictions and crystal structures are summarised in Additional file 10: Table S4. For illustration, identified permissive stretches were mapped onto the surface representation of the crystal structures of TEM1 β-lactamase (PDB 1AXB) (d), TpiA (shown as dimer, PDB 1TRE) (e), and the F1 part of ATP synthase (PDB 3OAA) (i). α-subunits (AtpA) are shown in *light green*, β-subunits are shown in *dark green*, and the γ-subunit is shown in *yellow*. PSs are marked in *grey*. For permissive site numbering refer to Additional file 3: Figure S1; Additional file 4: Figure S2, and Additional file 9: Figure S6. For TpiA and Bla, two different orientations are displayed to capture all PSs. Note that PSVIII within TpiA is not surface exposed and therefore not visible. PSI and PSVII within AtpA are not resolved in the crystal structure and therefore not given. (ZIP 13 kb)

Additional file 12: Figure S8. Whole proteome analysis of *E. coli*. Relative surface accessibilities of observed permissive stretches (*dark grey*) and a random shuffling of permissive stretches (*light grey*) are plotted. The Overlap of both distributions appears in *medium grey*. The relative surface area (RSA) of a given single amino acid residue is calculated as the predicted accessible surface area in the polypeptide chain, relative to the maximal possible exposure of that residue in the center of a tri-peptide flanked with either glycine or alanine. The RSA of a site is calculated as the geometric mean of the RSAs of its flanking residues (see Methods). The RSA of a predicted stretch is then calculated as the maximum RSA of its constituent sites’ RSAs. (PDF 6 kb)

Additional file 13: Figure S9. Structural context and cleavability. TEV-tagged protein variants were expressed from their natural promoters from the low copy plasmid pSEVA132, and crude lysate was incubated with TEV protease. Samples before and after cleavage were separated by SDS PAGE, blotted, and detected with specific antibodies: TpiA variants, eTag antibody; Adk and GpsA variants, 6xHis antibody. To roughly normalise the ratio of target protein and TEV protease, 10× more concentrated lysate was used for GpsA variants to account for the low abundance of GpsA in the cytosol. (a) TpiAE55.1–.3 with variations in the TEV-tag sequence (Table 1); (b) TpiAN69; (c) TpiAT130.1 and .2 with variations in the TEV-tag sequence (Table 1); (d) TpiAT152; (e) GpsAC49 and P60, both insertions are located in the same secondary structural element; (f) GpsAM99; (g) GpsAI132; (h) GpsAQ269. (PNG 347 kb)

Additional file 14: Figure S10. Control: stabilisation of nucleotide pool in CFX. Time course of nucleotide inter-conversion of CFX derived from wild-type strain Ec with or without pre-treatment by TEV protease. Nucleotide concentrations were quantified at indicated time points by HPLC in triplicates. Values are means (*n* = 3) ± SD. The 95% confidence interval for fitting half-life is highlighted in *grey*. (PNG 33 kb)

Additional file 15: Figure S11. Cleavability of α- and β-subunits of F1 part of ATP synthase. Plasmid encoded versions of Strep and TEV-tagged variants of (a) AtpAH123 and 6xHis and TEV-tagged variants of (b) AtpDE101 before and after addition of TEV protease. Proteins were detected by either an anti-Strep or anti-6xHis antibody on a western blot. (PNG 23 kb)

Additional file 16: Table S5. Plasmids used in this study. (DOCX 136 kb)

Additional file 17: Table S6. Primers used in this study. (DOCX 164 kb)

## Abbreviations

Adk: Adenylate kinase
ADP: Adenosine diphosphate
AMP: Adenosine monophosphate
ASA: Absolute surface area
aTc: Anhydrotetracycline
ATP: Adenosine triphosphate
Bla: β-lactamase
CFX: Cell-free extract
CoS-MAGE: Co-selection Multiplex automated genome engineering
DHAP: Dihydroxyacetone phosphate
DNA: Deoxyribonucleic acid
DTT: Dithiothreitol
GpsA: Glycerol-3-phosphate dehydrogenase
HPLC: High-performance liquid chromatography
HSP: High-scoring pair
IPTG: Isopropyl β-D-1-thiogalactopyranoside
LB: Lysogeny broth
MAGE: Multiplex automated genome engineering
MBP: Maltose binding protein
MSA: Multiple sequence alignment
NMP: Nucleoside monophosphate
NMR: Nuclear magnetic resonance
NTP: Nucleoside triphosphate
PCR: Polymerase chain reaction
PDB: Protein Data Bank
PS: Permissive stretch
PSS: Permissive stretch search
RSA: Relative surface accessibility
SD: Standard deviation
Ser: Serine
SS: Secondary structure
TEV: Tobacco etch virus
TEV-tag: TEV protease recognition site
Thr: Threonine
TpiA: Triosephosphate isomerase
WT: Wild type
X-gal: 5-Bromo-4-chloro-indolyl-β-D-galactopyranoside
