# Supplementary material for: Sequence-based prediction of permissive stretches for internal protein tagging and knockdown
Source: BMC Biol. 2017 Oct 30;15:100. doi: 10.1186/s12915-017-0440-0 (PMC5661948; doi:10.1186/s12915-017-0440-0)
Supplement: Supplementary file 1 — Mapping of known permissive sites within TpiA and Bla. A known permissive site (first column, given as residue number) within TpiA and Bla [32] was assigned to a predicted stretch (PS) if it mapped directly within a PS (see Additional file 3: Figure S1 for illustration) or if it was shifted by few residues but at least mapped to the same secondary structural element (“scattered” permissive sites). The number of residues by which a given “scattered” site is shifted is given in column 5. Note that, due to the experimental approach, which selected for only functional protein variants, there are no known non-permissive sites for TpiA. (DOCX 90 kb) [file 12915_2017_440_MOESM1_ESM.docx]

| Permissive site (residue) | Assigned to predicted stretch # ^a^ | Maps within same secondary structural element of PS? | Maps directly within PS? | Scatters around PS? +/- number of residues |
| --- | --- | --- | --- | --- |
| **TpiA** | | | | |
| N25 | PSI | yes/helix | no | PSI + 3 |
| E55 | PSIII | yes/helix | yes | / |
| K117 | PSV | yes/helix | yes | / |
| E118 | PSV | yes/helix | no | PSV + 1 |
| V142 | PSVI | yes/helix | yes | / |
| V150 | PSVI | yes/helix | yes | / |
| T153 | PSVI | yes/helix | yes | / |
| A157 | no | no | no | / |
| F159 | no | no | no | / |
| E160 | no | no | no | / |
| A195 | PSVII | yes/helix | yes | / |
| A199 | PSVII | yes/helix | yes | / |
| N200 | PSVII | yes/helix | yes | / |
| K247 | PSVIII | yes/helix | yes | / |
| E250 | PSVIII | yes/helix | no | PSVIII + 3 |
| A251 | PSVIII | yes/helix | no | PSVIII + 4 |
| **Non-permissive sites** |  |  |  |  |
| not available |  |  |  |  |
| **Bla** | | | | |
| **Permissive site (residue)** |  |  |  |  |
| E195 (197)* | PSVI | yes/coil | yes | / |
| L196 (198) | PSVI | yes/coil | yes | / |
| T261 (265) | PSIX | yes/coil | no | PSIX-5 |
| A266 (270) | PSIX | yes/helix | yes | / |
| M268 (272) | PSIX | yes/helix | yes | / |
| **Semi-permissive sites** |  |  |  |  |
| E35 (37) | no | no | / | / |
| Y95 (97) | PSIII | yes/sheet | yes | / |
| Q97 (99) | PSIII | yes/coil | yes | / |
| V117 (119) | no | no | / | / |
| Q204 (206) | no | no | / | / |
| V214 (216) | PSVII | yes/coil | yes | / |
| G216 (218) | PSVII | yes/coil | yes | / |
| I256 (260) | no | no | / | / |
| Y260 (264) | no | no | / | / |
| N272 (276) | no | no | / | / |
| **Non-permissive sites** |  |  |  |  |
| E61 (63) | no | no | / | / |
| M66 (68) | no | no | / | / |
| D161 (163) | no | no | / | / |
| R162 (164) | no | no | / | / |
| I206 (208) | no | no | / | / |
| A230 (232) | no | no | / | / |
| D231 (233) | no | no | / | / |
| I244(246) | no | no | / | / |
