# Supplementary figures and images for: Sequence-based prediction of permissive stretches for internal protein tagging and knockdown
Source: BMC Biol. 2017 Oct 30;15:100. doi: 10.1186/s12915-017-0440-0 (PMC5661948; doi:10.1186/s12915-017-0440-0)

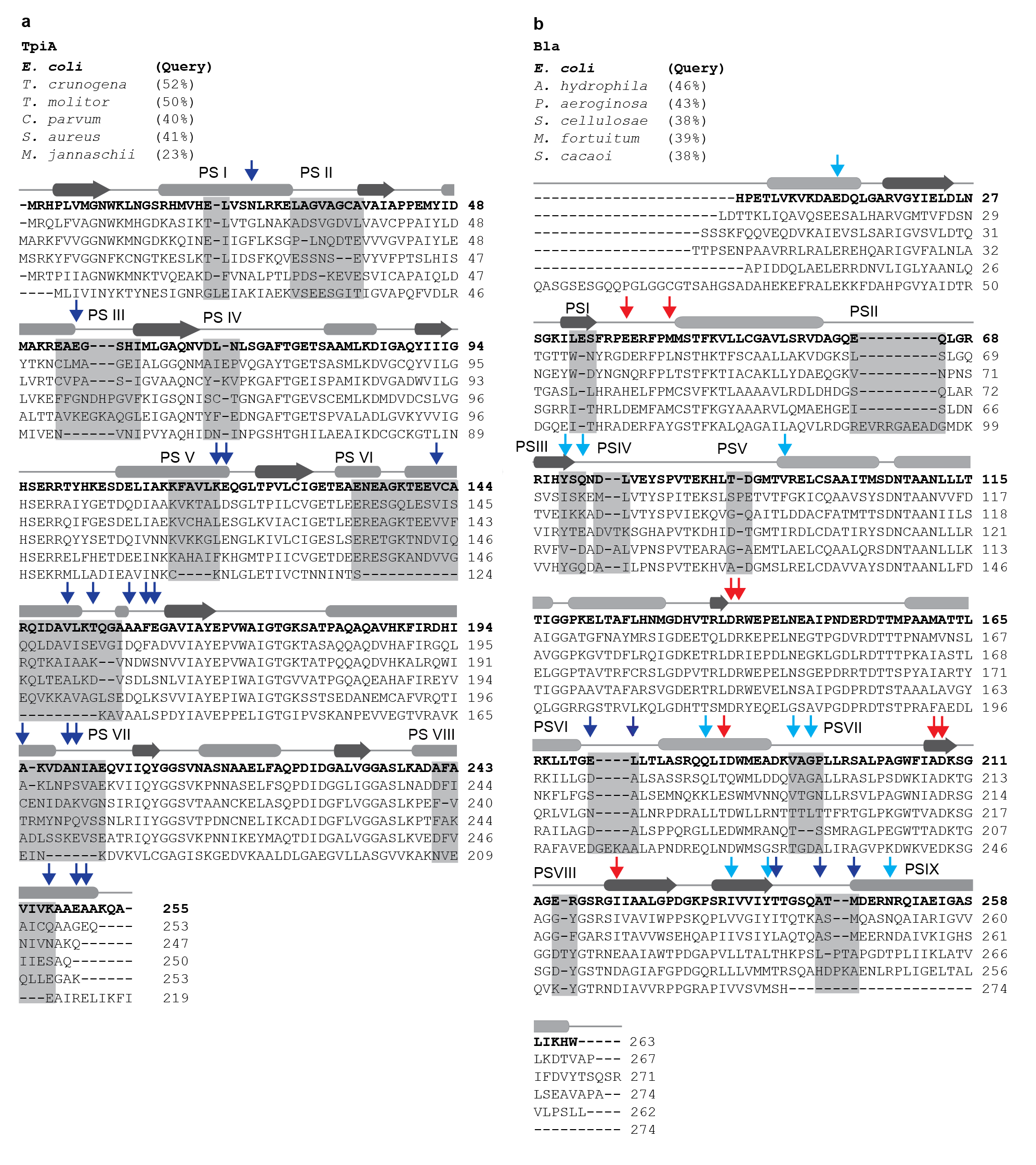

Supplement: Supplementary file 3 — Mapping of previously known permissive sites within TpiA and Bla onto the corresponding MSA. (a) E. coli triosephosphate isomerase (TpiA), (b) E. coli TEM1 β-lactamase [32]. Known permissive sites are summarised in Additional file 1: Table S1. Predicted permissive stretches (PSs) are highlighted in grey. Dark blue arrows functional permissive sites, light blue arrows semi-permissive sites (sequence insertion altered the function to some extent), red arrows non-permissive sites. Predicted secondary structure is given above the alignment. Light grey boxes depict α-helices, dark grey arrows depict β-strands, and the grey line depicts unstructured coils. Note that due to the experimental approach, which selected for only functional protein variants, there are no known non-permissive sites for TpiA. (PNG 429 kb) [file 12915_2017_440_MOESM3_ESM.png]

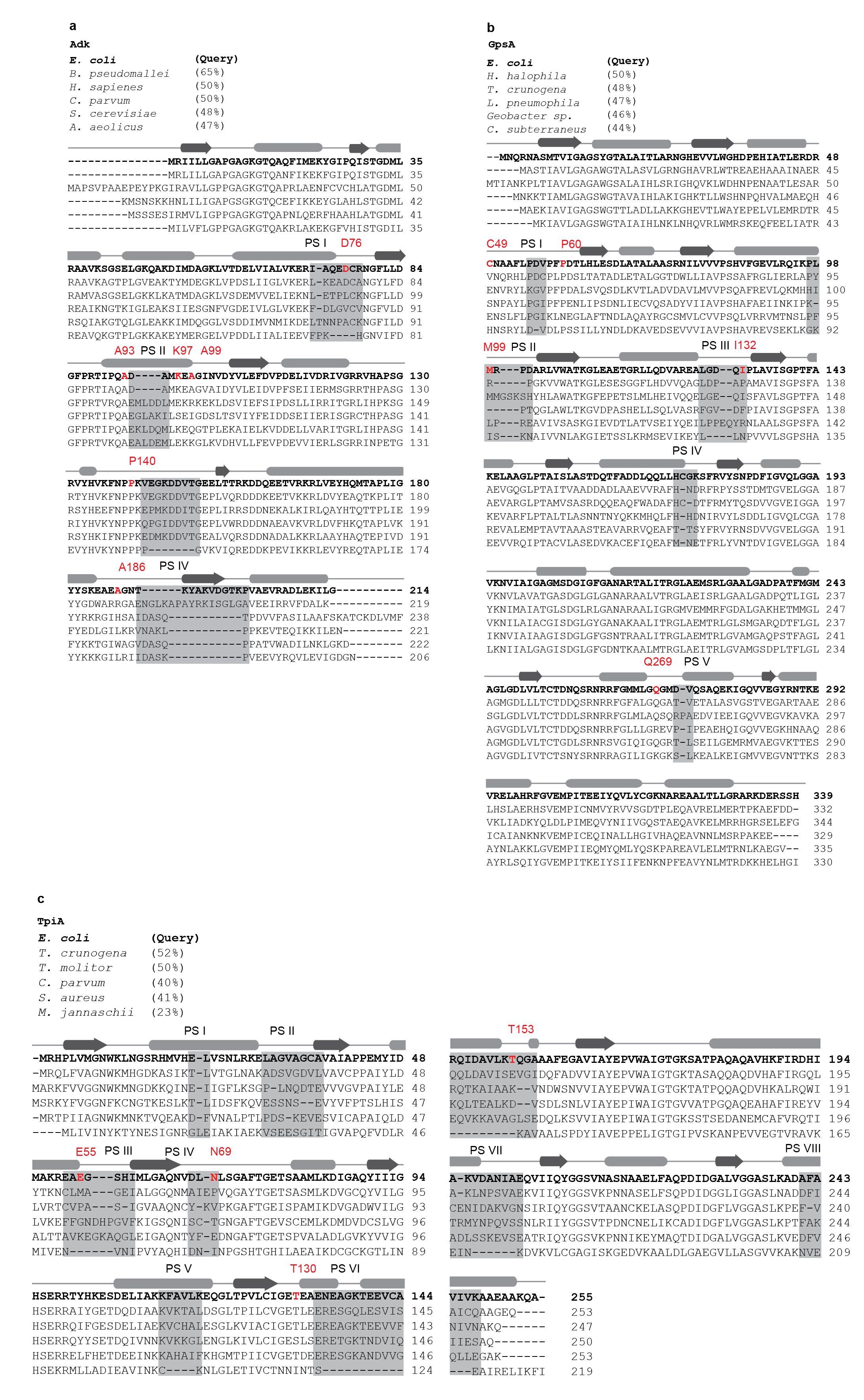

Supplement: Supplementary file 4 — Identification of permissive stretches in Adk, GpsA, and TpiA. MSAs for Adk (a) GpsA (b), and TpiA (c). Identified permissive stretches are highlighted in grey. Residues which were chosen for TEV-tag insertion are given as red numbers above the alignment. The predicted secondary structure for both proteins is given above the alignments. Light grey boxes indicate α-helices, dark grey depict β-strands. (PNG 631 kb) [file 12915_2017_440_MOESM4_ESM.png]

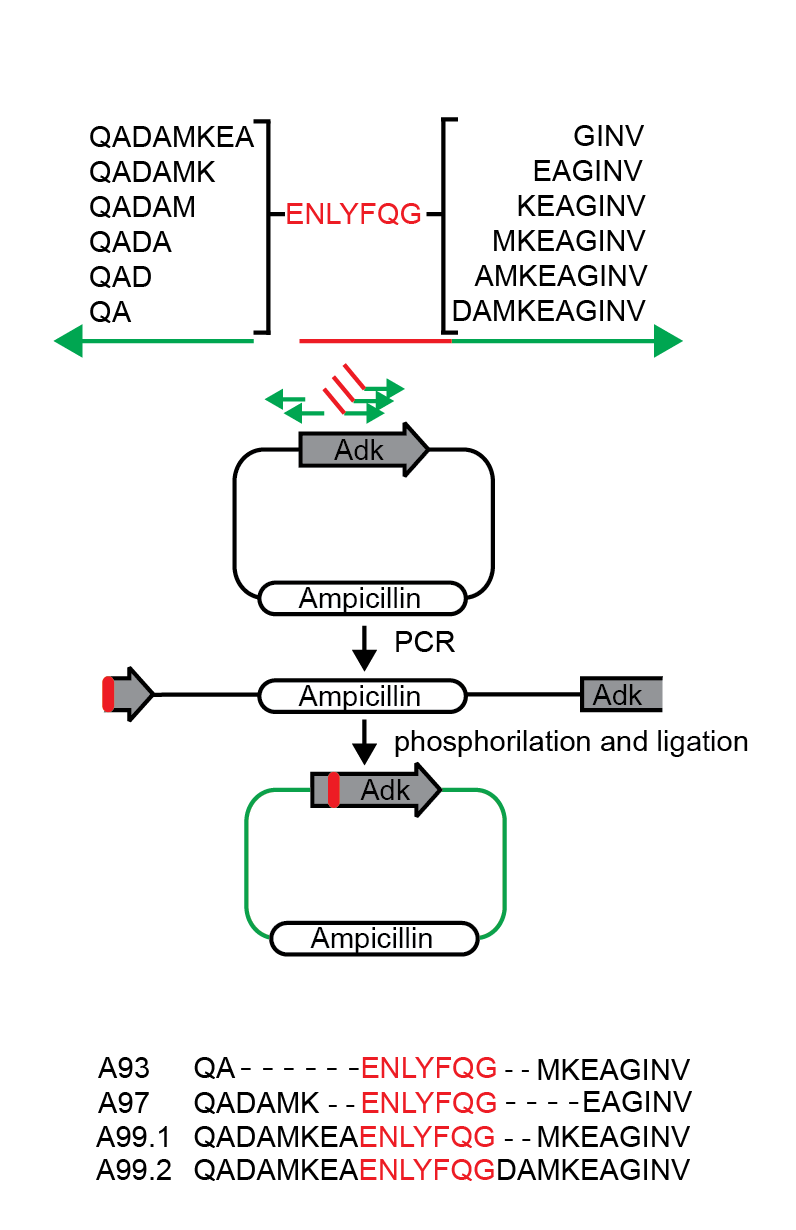

Supplement: Supplementary file 5 — Construction of Adk test library. A TEV-tag insertion library around PSII of Adk was generated by vector PCR using a pool of different primers. The primer design allowed for different insert designs such as simple insertion, replacements, and duplications. We sampled the region, spanning residues A93 to A99 for potential insertions. The identified gap in our alignment spans D94 and A95 of E. coli Adk. Sequences of four clones are given, which were selected for further analysis. (PNG 58 kb) [file 12915_2017_440_MOESM5_ESM.png]

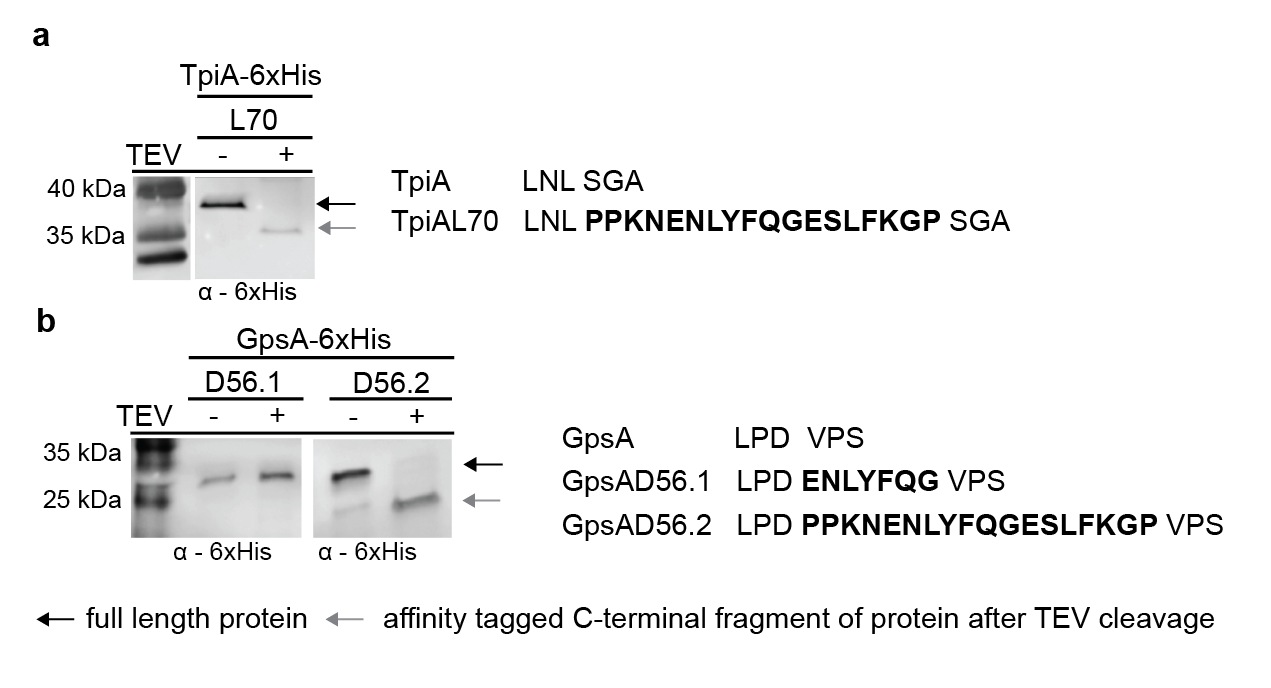

Supplement: Supplementary file 6 — Engineering cleavable TpiA and GpsA variants using an extended TEV-tag. (a) TpiAL70 is located in a 15-residue loop (Additional file Additional file 4: Figure S2c and Additional file Additional file 11: Figure S7c). An extended TEV-tag flanked by C- and N-terminal extensions derived from the TEV polyprotein was inserted after position L70. Hydrolysis of the variant TpiAL70 by TEV protease was tested as described for Additional file Additional file 13: Figure S9. Note that Additional file Additional file 13: Figure S9b verifies that the minimal TEV-tag inserted after residue N69 is not cleaved by TEV protease. TpiA was C-terminally 6xHis-tagged and detected with a 6xHis-specific antibody. (b) GpsAD56 is located in a 16-residue loop, as predicted by secondary structure prediction (Additional file Additional file 4: Figure S2b and Additional file Additional file 11: Figure S7h). The minimal TEV-tag as well as an extended TEV-tag flanked by C- and N-terminal extensions derived from the TEV polyprotein were inserted after position D57. Hydrolysis of the corresponding variants GpsAD57.1 and GpsAD57.2 by TEV protease was tested as described for Additional file Additional file 13: Figure S9. GpsA was C-terminally 6xHis-tagged and detected with a 6xHis-specific antibody. (PNG 83 kb) [file 12915_2017_440_MOESM6_ESM.png]

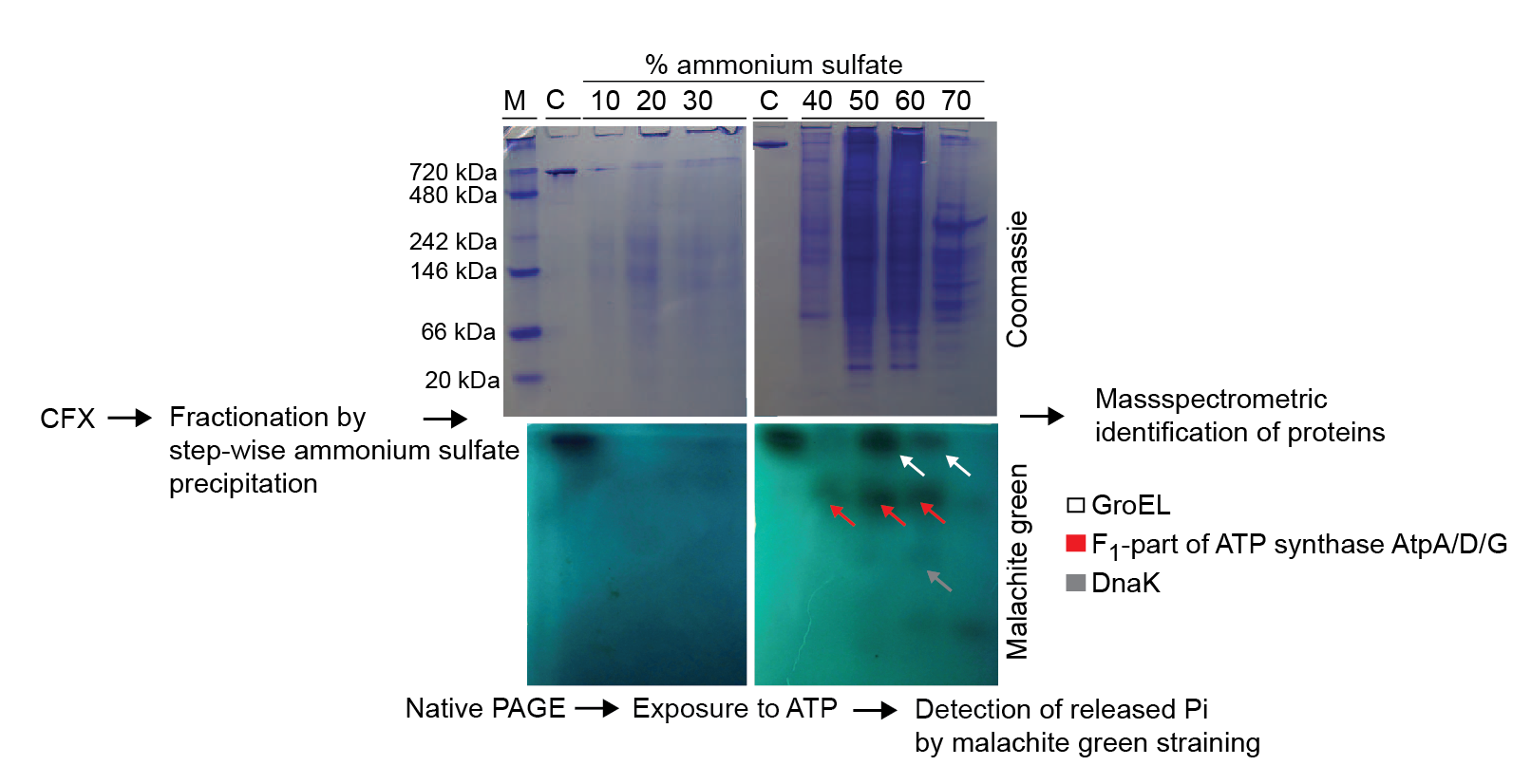

Supplement: Supplementary file 8 — Systematic identification of ATP sinks in CFX. CFX was fractionated by step-wise (increments of 10% per step) ammonium sulphate precipitation. Fractions were separated by native PAGE. The gel was subsequently incubated in ATP and stained to detect liberated inorganic phosphate (Pi) by malachite green as described [67] (lower panel). In brief: The gel was dipped for 30 min in redox buffer (30 mM Tris/HCl, 80 mM KCl, 5 mM MgCl2 10 mM DTT), followed by 1 h incubation in 10 mL substrate buffer (30 mM Tris, 80 mM KCl, 5 mM MgCl2, 10 mM ATP) at 37 °C. We added 2 mL of a malachite green solution (1.2 mL 0.44 mg malachite green in 100 mL H2O/H2SO4 and 0.8 mL 7.5% ammonium molybdate) directly to the substrate buffer. Colour development was allowed to proceed for 1 h. Samples were split after fractionation and additionally with Coomassie Blue to visualise all present protein bands (upper panel). Dark green spots indicate the presence of ATPase activity. Spots were cut from the gel and proteins were identified by mass spectrometry. White arrows spots corresponding to GroEL, red arrows spots corresponding to the F1 part of ATP synthase grey arrow spot corresponding to DnaK; purified GroEL was used as a positive control (C). (PNG 344 kb) [file 12915_2017_440_MOESM8_ESM.png]

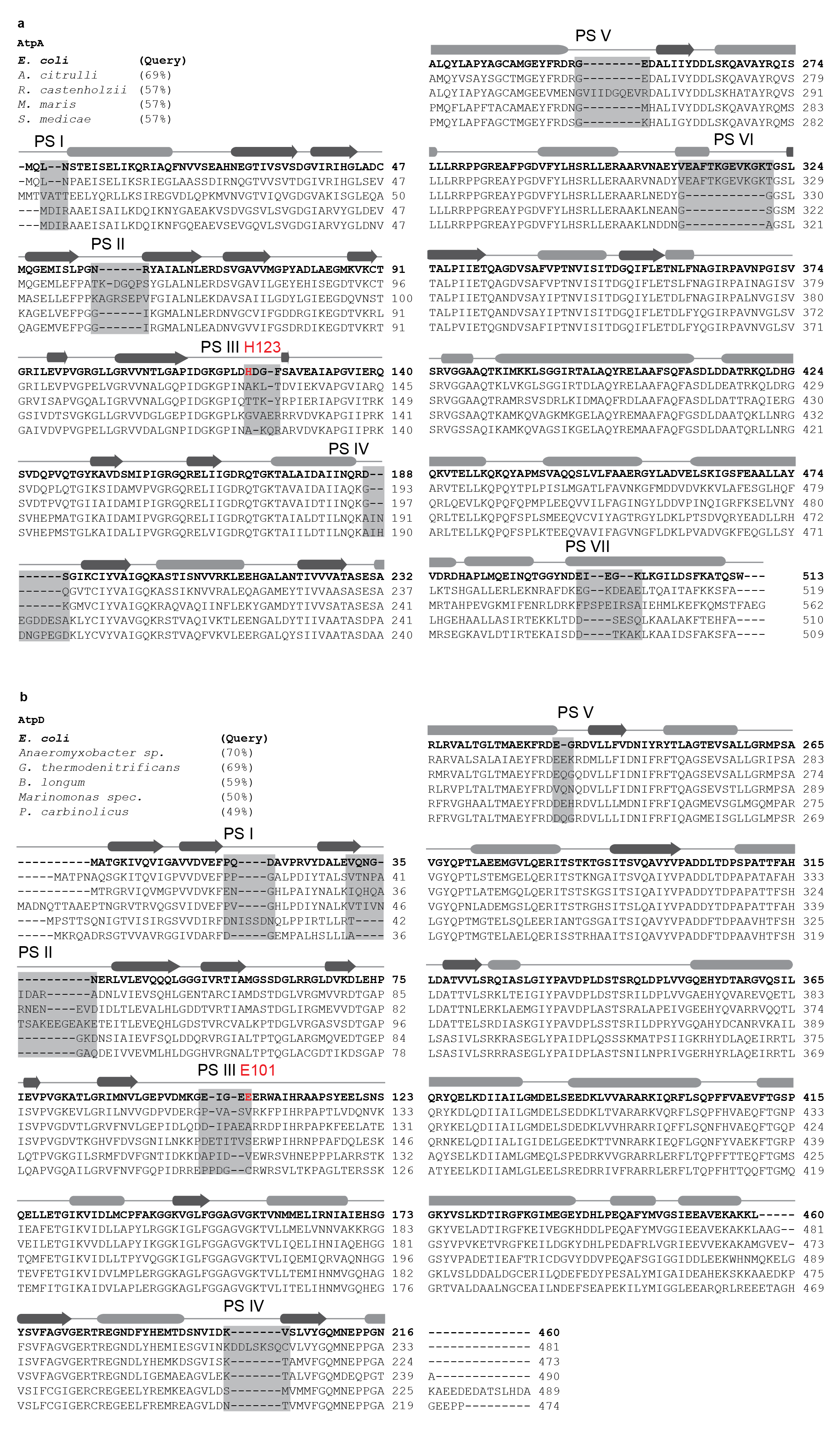

Supplement: Supplementary file 9 — Identification of permissive stretches in the α- and β-subunits of Fo part of ATP synthase. MSAs were generated for AtpA (a) and AtpD (b). Permissive stretches are highlighted in grey. Insertion positions are given in red. The predicted secondary structure for both proteins is given above the alignments. Light grey boxes indicate α-helices, dark grey arrows indicate β-strands. (PNG 613 kb) [file 12915_2017_440_MOESM9_ESM.png]

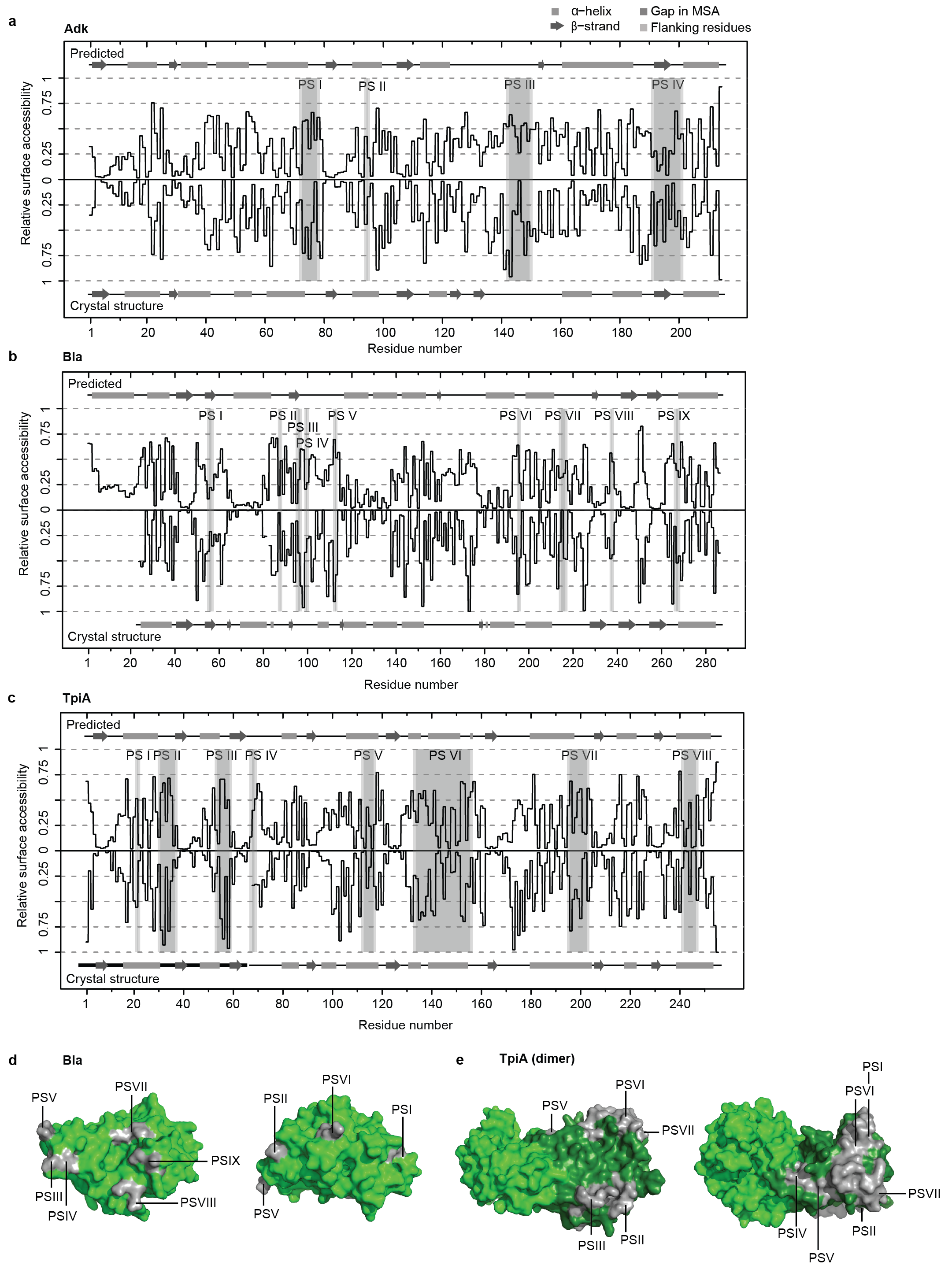

Supplement: Supplementary file 11 — PSS-identified permissive stretches are enriched to be surface accessible. Each plot summarizes the predicted surface accessibility and the predicted secondary structure of each residue for all test proteins (upper panel within each plot) as well as relative surface accessibility and secondary structure calculated from available crystal structures by the DSSP algorithm (lower panel within each plot). (a) Adk, (b) Bla, (c) TpiA, (f) AtpA, (g) AtpD, and (h) GpsA. PSs are highlighted in grey. The RSA of a site is defined as the geometric mean of the RSAs of its flanking residues, while the RSA of a predicted stretch is defined as the maximum site RSA in the stretch. RSAs of stretches derived from predictions and crystal structures are summarised in Additional file 10: Table S4. For illustration, identified permissive stretches were mapped onto the surface representation of the crystal structures of TEM1 β-lactamase (PDB 1AXB) (d), TpiA (shown as dimer, PDB 1TRE) (e), and the F1 part of ATP synthase (PDB 3OAA) (i). α-subunits (AtpA) are shown in light green, β-subunits are shown in dark green, and the γ-subunit is shown in yellow. PSs are marked in grey. For permissive site numbering refer to Additional file 3: Figure S1; Additional file 4: Figure S2, and Additional file 9: Figure S6. For TpiA and Bla, two different orientations are displayed to capture all PSs. Note that PSVIII within TpiA is not surface exposed and therefore not visible. PSI and PSVII within AtpA are not resolved in the crystal structure and therefore not given. (ZIP 13 kb) [file 12915_2017_440_MOESM11_ESM.zip › Additional file 11 - Figure S7-abcde.png]

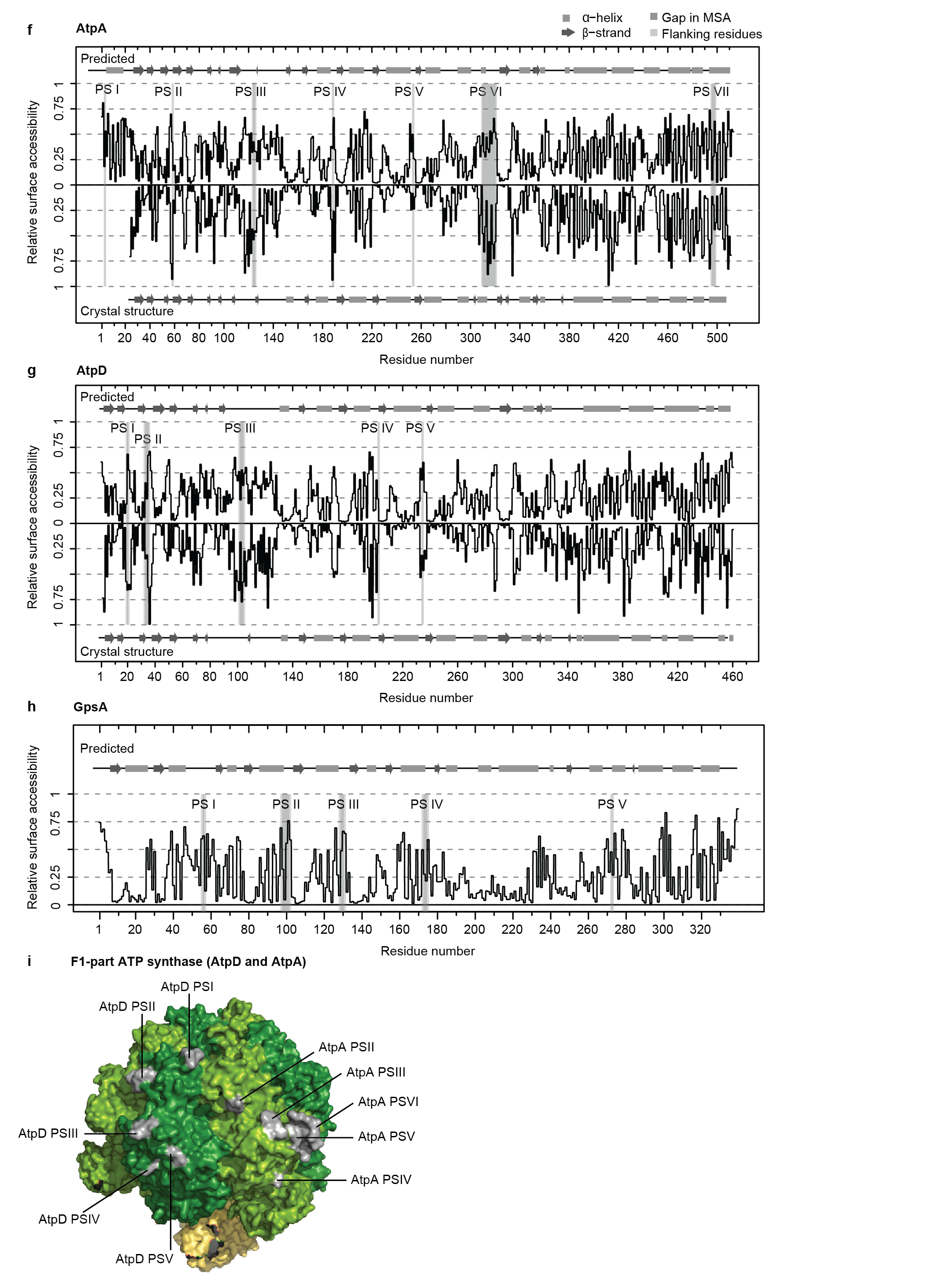

Supplement: Supplementary file 11 — PSS-identified permissive stretches are enriched to be surface accessible. Each plot summarizes the predicted surface accessibility and the predicted secondary structure of each residue for all test proteins (upper panel within each plot) as well as relative surface accessibility and secondary structure calculated from available crystal structures by the DSSP algorithm (lower panel within each plot). (a) Adk, (b) Bla, (c) TpiA, (f) AtpA, (g) AtpD, and (h) GpsA. PSs are highlighted in grey. The RSA of a site is defined as the geometric mean of the RSAs of its flanking residues, while the RSA of a predicted stretch is defined as the maximum site RSA in the stretch. RSAs of stretches derived from predictions and crystal structures are summarised in Additional file 10: Table S4. For illustration, identified permissive stretches were mapped onto the surface representation of the crystal structures of TEM1 β-lactamase (PDB 1AXB) (d), TpiA (shown as dimer, PDB 1TRE) (e), and the F1 part of ATP synthase (PDB 3OAA) (i). α-subunits (AtpA) are shown in light green, β-subunits are shown in dark green, and the γ-subunit is shown in yellow. PSs are marked in grey. For permissive site numbering refer to Additional file 3: Figure S1; Additional file 4: Figure S2, and Additional file 9: Figure S6. For TpiA and Bla, two different orientations are displayed to capture all PSs. Note that PSVIII within TpiA is not surface exposed and therefore not visible. PSI and PSVII within AtpA are not resolved in the crystal structure and therefore not given. (ZIP 13 kb) [file 12915_2017_440_MOESM11_ESM.zip › Additional file 11 - Figure S7-fghi.png]

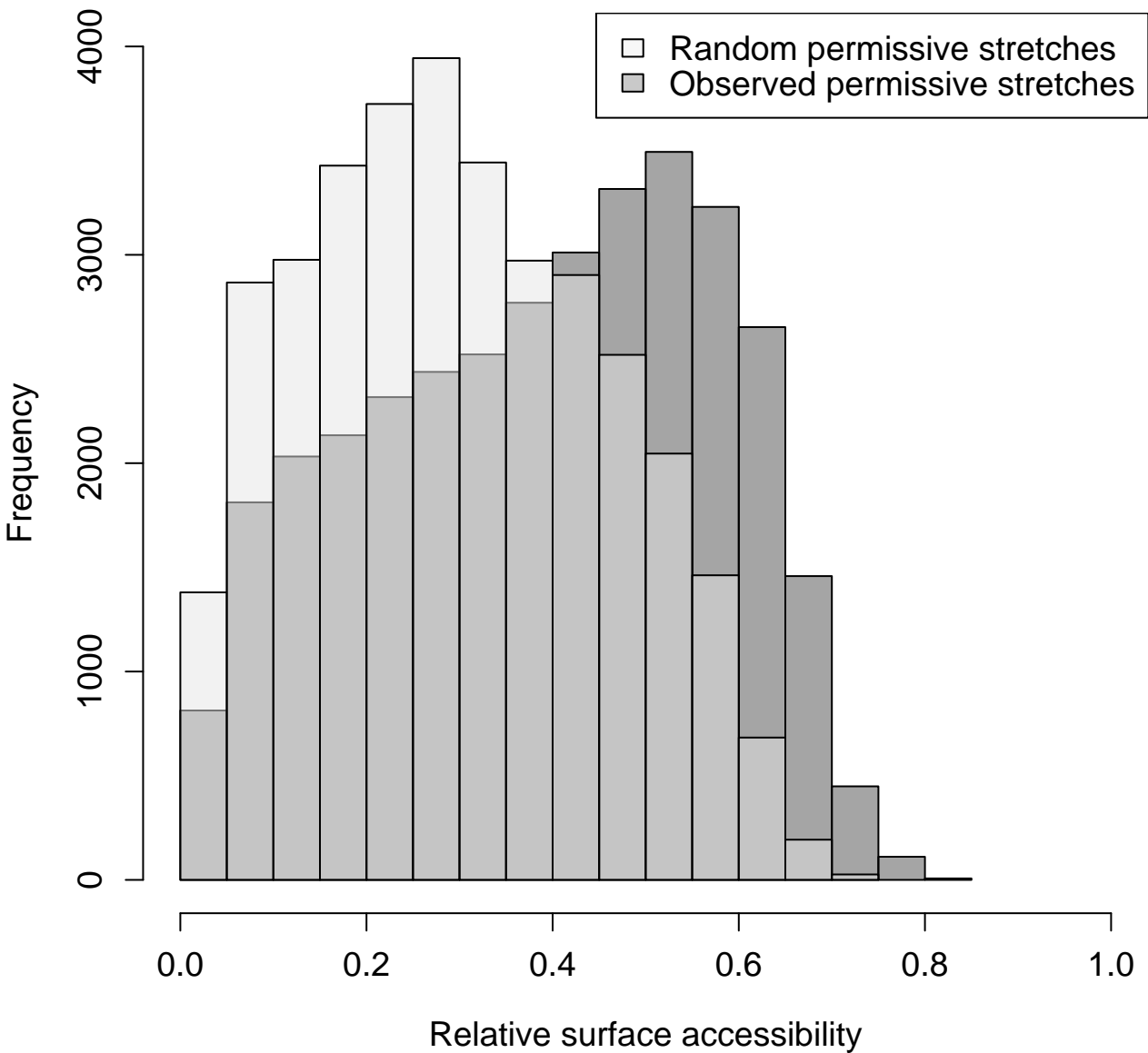

Supplement: Supplementary file 12 — Whole proteome analysis of E. coli. Relative surface accessibilities of observed permissive stretches (dark grey) and a random shuffling of permissive stretches (light grey) are plotted. The Overlap of both distributions appears in medium grey. The relative surface area (RSA) of a given single amino acid residue is calculated as the predicted accessible surface area in the polypeptide chain, relative to the maximal possible exposure of that residue in the center of a tri-peptide flanked with either glycine or alanine. The RSA of a site is calculated as the geometric mean of the RSAs of its flanking residues (see Methods). The RSA of a predicted stretch is then calculated as the maximum RSA of its constituent sites’ RSAs. (PDF 6 kb) [file 12915_2017_440_MOESM12_ESM.pdf]

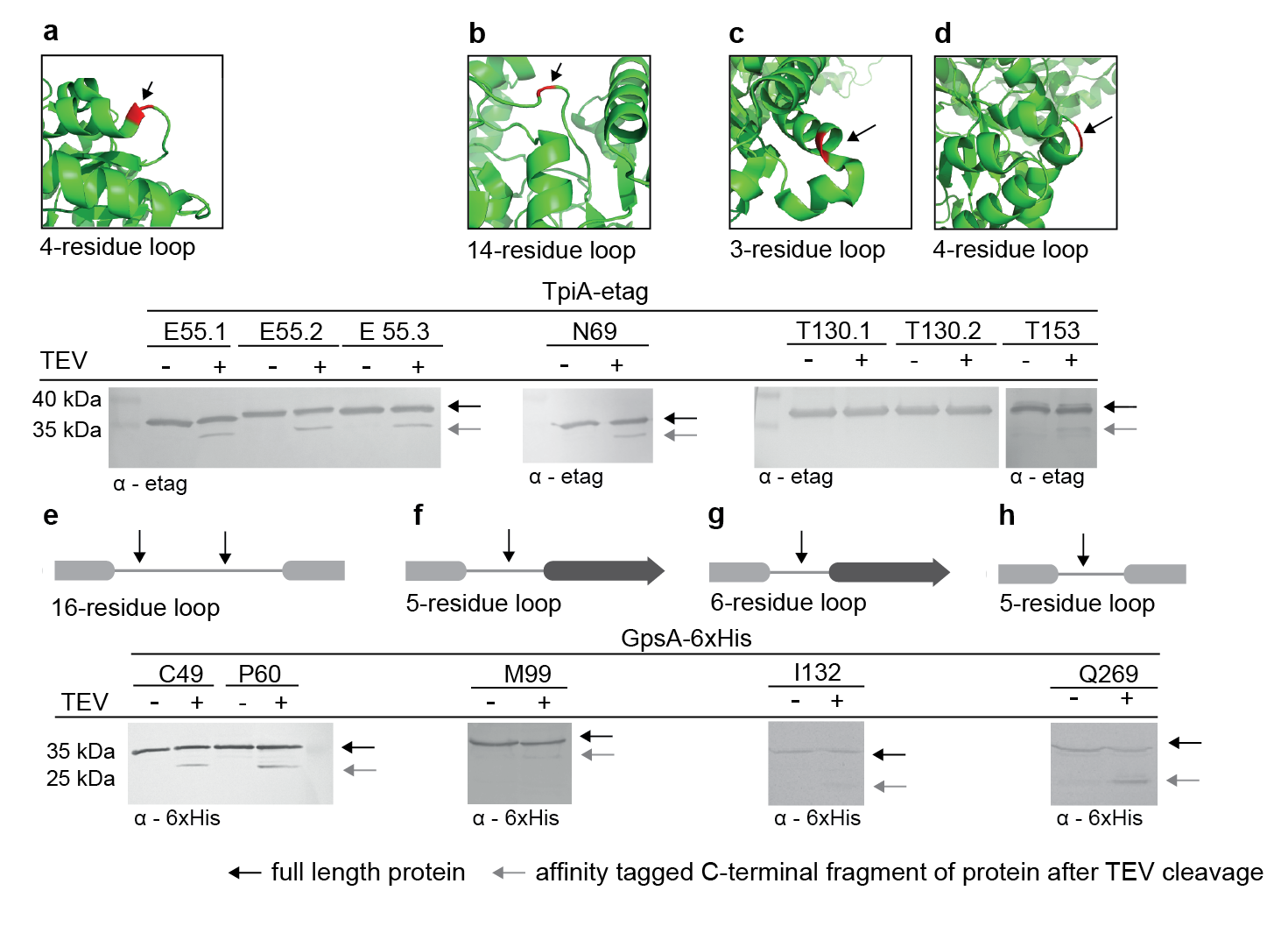

Supplement: Supplementary file 13 — Structural context and cleavability. TEV-tagged protein variants were expressed from their natural promoters from the low copy plasmid pSEVA132, and crude lysate was incubated with TEV protease. Samples before and after cleavage were separated by SDS PAGE, blotted, and detected with specific antibodies: TpiA variants, eTag antibody; Adk and GpsA variants, 6xHis antibody. To roughly normalise the ratio of target protein and TEV protease, 10× more concentrated lysate was used for GpsA variants to account for the low abundance of GpsA in the cytosol. (a) TpiAE55.1–.3 with variations in the TEV-tag sequence (Table 1); (b) TpiAN69; (c) TpiAT130.1 and .2 with variations in the TEV-tag sequence (Table 1); (d) TpiAT152; (e) GpsAC49 and P60, both insertions are located in the same secondary structural element; (f) GpsAM99; (g) GpsAI132; (h) GpsAQ269. (PNG 347 kb) [file 12915_2017_440_MOESM13_ESM.png]

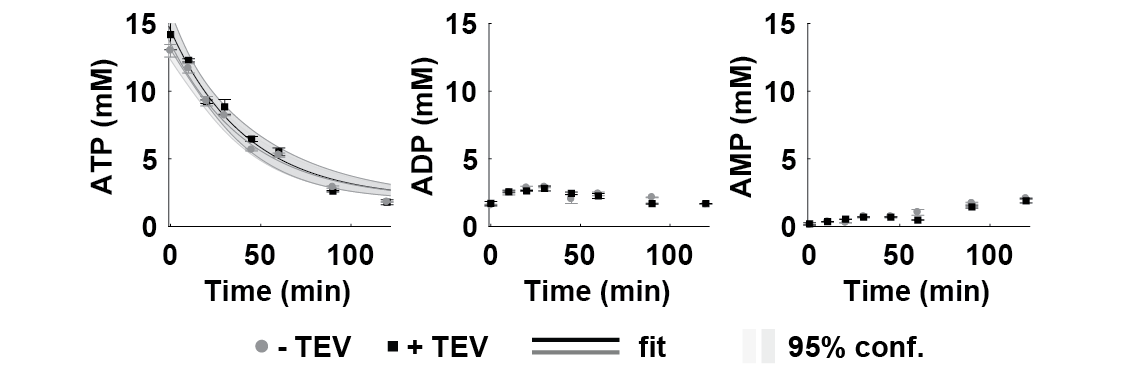

Supplement: Supplementary file 14 — Control: stabilisation of nucleotide pool in CFX. Time course of nucleotide inter-conversion of CFX derived from wild-type strain Ec with or without pre-treatment by TEV protease. Nucleotide concentrations were quantified at indicated time points by HPLC in triplicates. Values are means (n = 3) ± SD. The 95% confidence interval for fitting half-life is highlighted in grey. (PNG 33 kb) [file 12915_2017_440_MOESM14_ESM.png]

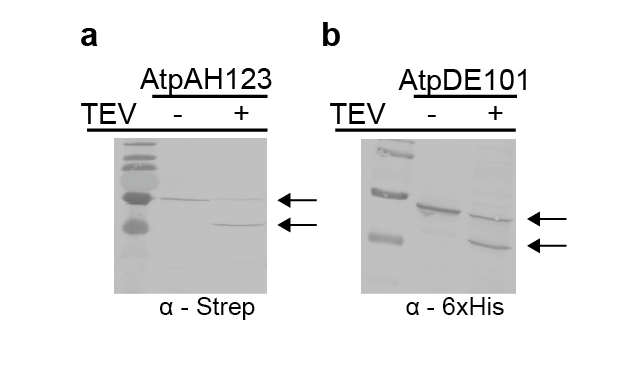

Supplement: Supplementary file 15 — Cleavability of α- and β-subunits of F1 part of ATP synthase. Plasmid encoded versions of Strep and TEV-tagged variants of (a) AtpAH123 and 6xHis and TEV-tagged variants of (b) AtpDE101 before and after addition of TEV protease. Proteins were detected by either an anti-Strep or anti-6xHis antibody on a western blot. (PNG 23 kb) [file 12915_2017_440_MOESM15_ESM.png]
