# Supplementary material for: Sequence-based prediction of permissive stretches for internal protein tagging and knockdown
Source: BMC Biol. 2017 Oct 30;15:100. doi: 10.1186/s12915-017-0440-0 (PMC5661948; doi:10.1186/s12915-017-0440-0)
Supplement: Supplementary file 7 — Strains used in this study. (DOCX 20 kb) [file 12915_2017_440_MOESM7_ESM.docx]

| **Strains** | **Genotype/Description** | **Reference** |
| --- | --- | --- |
| DH10B | *F- endA1 recA1 galE15 galK16 nupG rpsL ΔlacX74 Φ80lacZΔM15 araD139 Δ(ara,leu)7697 mcrA Δ(mrr-hsdRMS-mcrBC) λ-* | Internal strain collection |
| BL21 | *gal hsdS B ompT* | Internal strain collection |
| EcNR 1 | MG1655 Δ(*ybhB*-*bioAB*)::[λcI857 N(*cro*-*ea59*)::*tetR*-*bla*] | Addgene #26930 |
| Ec | EcNR1 translational knockout of mutS according to ref | This study |
| W3110 *adk::kan* [pCOMP-*adk* pP_BAD_-ISceI] | Adk-replacement system: W3110 with *adk* replaced by a kanamycin cassette, plasmid pCOMP-adk can be eliminated by induction of I-SceI nuclease expression from plasmid pPBAD-ISceI | [1] |
| W3110 *secBgpsA::kan* [pCOMP-*secBgpsA* pP_BAD_-ISceI] | GpsA-replacement system: W3110 with *secB and gpsA* replaced by a kanamycin cassette, plasmid pCOMP-*secBgpsA* can be eliminated by induction of I-SceI nuclease expression from plasmid pPBAD-ISceI | [1] |
| W3110 *groE::kan* [pCOMP-*groE* pP_BAD_-ISceI] | GroEL-replacement system: W3110 with *groE*  replaced by a kanamycin cassette, plasmid pCOMP-*groE* can be eliminated by induction of I-SceI nuclease expression from plasmid pPBAD-ISceI | [1] |
| *tpiA*::FRT *amn*::FRT | Derivative of KEIO strain BW25113 tpiA::kan with the kanamycin resistance gene eliminated, followed by subsequent deletion of *amn* by P1 transduction using lysate of KEIO strain BW25113 amn::kan and followed by elimination of the kanamycin resistance cassette | This study |
| *EcΔatpA* | KEIO strain BW25113 *atpA*::kan | [2] |
| *EcΔatpD* | KEIO strain BW25113 *atpD*::kan | [2] |
| EcAtpAH123 | Chromosomal integration of the switchable *atpAH123* variant on the genome of Ecwt by MAGE; *rpSL(*-) phenotype due to co-selection | This study |
| EcAtpDE101 | Chromosomal integration of the switchable *atpDE101* variant on the genome of Ecwt by MAGE; *rpSL(*-) phenotype due to co-selection | This study |
| EcAdkD76.3 | Chromosomal integration of the switchable *adk76.1*  variant on the genome of Ecwt by MAGE | This study |
| Ec* | Chromosomal integration of the translational knockout for *amn* on the genome of Ecwt by MAGE | This study |
| EcTpiAstop* | Chromosomal integration of the double translational knockout for *amn* and *tpiA* on the genome of Ecwt by MAGE | This study |
| EcTpiA L70* | Chromosomal integration of the switchable *tpiA70.1* variant on the genome of Ecwt*by MAGE; *malK*-phenotype due to co-selection | This study |

1. Billerbeck S, Panke S: **A genetic replacement system for selection-based engineering of essential proteins**. *Microb Cell Fact* 2012, **11**(1):110.

2. Baba T, Ara T, Hasegawa M, Takai Y, Okumura Y, Baba M, Datsenko KA, Tomita M, Wanner BL, Mori H: **Construction of Escherichia coli K-12 in-frame, single-gene knockout mutants: the Keio collection**. *Molecular systems biology* 2006, **2**:2006 0008.
