# Supplementary material for: Sequence-based prediction of permissive stretches for internal protein tagging and knockdown
Source: BMC Biol. 2017 Oct 30;15:100. doi: 10.1186/s12915-017-0440-0 (PMC5661948; doi:10.1186/s12915-017-0440-0)
Supplement: Supplementary file 10 — Structural features of predicted permissive stretches determined by structure prediction and crystal structures. Secondary structure (SS) and RSA were predicted from sequence using NetSurfP [45] and calculated from crystal structures using the DSSP algorithm [63]. A crystal structure for GpsA was not available. The RSA of a stretch is defined as the maximum RSA of its constituent sites. The correlations of RSA and SS between predictions and crystal structures at all residues were computed by distance correlation [65]. The crystal structure was not resolved in TpiA PSIV and AtpA PSI. See Methods for more precise definitions of these calculations. (DOCX 68 kb) [file 12915_2017_440_MOESM10_ESM.docx]

| **Protein**  UniProtKB  PDB | **PS** | **NetSurfP** | **DSSP** | **RSA Correlation** | **SS Correlation** |
| --- | --- | --- | --- | --- | --- |
| **Adk**  P69441  1AKE | I | 0.58 | 0.61 | 0.74 | 0.7 |
|  | II | 0.3 | 0.27 |  |  |
|  | III | 0.58 | 0.82 |  |  |
|  | IV | 0.52 | 0.43 |  |  |
| **TpiA**  P0A858  1TRE | I | 0.17 | 0.23 | 0.8 | 0.8 |
|  | II | 0.62 | 0.85 |  |  |
|  | III | 0.7 | 0.76 |  |  |
|  | IV | 0.45 | NA |  |  |
|  | V | 0.32 | 0.24 |  |  |
|  | VI | 0.67 | 0.67 |  |  |
|  | VII | 0.59 | 0.62 |  |  |
|  | VIII | 0.32 | 0.06 |  |  |
| **GpsA**  P0A6S7  N/A | I | 0.61 |  |  |  |
|  | II | 0.67 |  |  |  |
|  | III | 0.65 |  |  |  |
|  | IV | 0.37 |  |  |  |
|  | V | 0.36 |  |  |  |
| **Bla**  P62593  1AXB | I | 0.21 | 0.29 | 0.82 | 0.79 |
|  | II | 0.26 | 0.11 |  |  |
|  | III | 0.43 | 0.56 |  |  |
|  | IV | 0.24 | 0.34 |  |  |
|  | V | 0.6 | 0.76 |  |  |
|  | VI | 0.41 | 0.62 |  |  |
|  | VII | 0.42 | 0.48 |  |  |
|  | VIII | 0.52 | 0.46 |  |  |
|  | IX | 0.42 | 0.49 |  |  |
| **AtpA**  P0ABB0  3OAA | I | 0.31 | NA | 0.69 | 0.77 |
|  | II | 0.25 | 0.37 |  |  |
|  | III | 0.38 | 0.49 |  |  |
|  | IV | 0.49 | 0.5 |  |  |
|  | V | 0.36 | 0.14 |  |  |
|  | VI | 0.58 | 0.7 |  |  |
|  | VII | 0.45 | 0.4 |  |  |
| **AtpD**  P0ABB4  3OAA | I | 0.6 | 0.63 | 0.69 | 0.74 |
|  | II | 0.69 | 0.79 |  |  |
|  | III | 0.44 | 0.67 |  |  |
|  | IV | 0.08 | 0 |  |  |
|  | V | 0.51 | 0.38 |  |  |
