# Supplementary material for: Sequence-based prediction of permissive stretches for internal protein tagging and knockdown
Source: BMC Biol. 2017 Oct 30;15:100. doi: 10.1186/s12915-017-0440-0 (PMC5661948; doi:10.1186/s12915-017-0440-0)
Supplement: Supplementary file 16 — Plasmids used in this study. (DOCX 136 kb) [file 12915_2017_440_MOESM16_ESM.docx]

| **Plasmid** | **Genotype/Construction/Comment** | **Reference/Source** |
| --- | --- | --- |
| **Parent plasmids** | | |
| pSEVA132 | pBBR1 ori, Amp^R^ | [1] |
| pCKO1 | pSC101 ori, Cm^R^ | [2] |
| pKTs | pUC ori, Amp^R^ | [3] |
| **Adk insertions** | | |
| pSEVA132-*adk* | pSEVA132 encoding for C-terminally 6xHis-tagged Adk under control of its natural promoter. *Adk* including its promoter were amplified from *E. coli* genomic DNA using primers *adk*-forward and *adk*-reverse and cloned into pSEVA132 via restriction sites *Xma*I and *Sac*I This construct was used as template for the insertion of various TEV cleavage sites. | [4] |
| pSEVA132-*adkD76* | All internal TEV-tag insertions were constructed by amplification and re-ligation of pSEVA132-adk using the forward and reverse primers for each specific insertion variant given in Supplementary Table 5. All forward primers were purchased as 5'-phosphorilated oligonucleotides. | This study |
| pSEVA132-*adkP140* | see pSEVA132-*adkD76* | This study |
| pSEVA132-*adkA186* | see pSEVA132-*adkD76* | This study |
| pSEVA132-*adkA93* | see pSEVA132-*adkD76* | This study |
| pSEVA132-*adkA93, K97, A99.1, A99.2* | constructed as outlined in Supplementary Figure 11 | This study |
| **GpsA insertions** | |  |
| pSEVA132-*secBgpsA* | pSEVA132 encoding for SecB and C-terminally 6xHis-tagged GpsA under control of the natural promoter of the operon. The natural *secB-gpsA* transcriptional unit was amplified with primers *secBgpsA*-forward and *secBgpsA*-reverse and cloned into pSEVA132 via restriction sites *PacI* and *Xma*I. This construct was used as template for the insertion of various TEV cleavage sites | [4] |
| pSEVA132-*secBgpsAC49* | All internal TEV-tag insertions were constructed by amplification and re-ligation of pSEVA132-*secBgpsA* using the forward and reverse primers for each specific insertion variant given in Supplementary Table 5. All reverse primers were purchased as 5'-phosphorilated oligonucleotides. | This study |
| pSEVA132-*secBgpsAP60* | see pSEVA132-*secBgpsAC49* | This study |
| pSEVA132-*secBgpsAM99* | see pSEVA132-secBgpsAC49 | This study |
| pSEVA132-*secBgpsAI132* | see pSEVA132-secBgpsAC49 | This study |
| pSEVA132-*secBgpsAQ269* | see pSEVA132-secBgpsAC49 | This study |
| pSEVA132-*secBgpsAD56.1* | Constructed as pSEVA132-secBgpsAC49, but Gibson assembly was used instead of re-ligation. | This study |
| pSEVA132-*secBgpsAD56.2* | see pSEVA132-secBgpsAD56.1 | This study |
| **TpiA insertions** | | |
| pCKO1-*tpiA* | pCKO1 encoding for C-terminally e-tagged *tpi*A under control of its natural promoter. This construct was used as template for the insertion of various TEV cleavage sites. *tpi*A and its promoter were amplified from *E. coli* chromosomal DNA and cloned into the MCS of pCKO1 via restriction sites *Xba*I and *Hin*dIII | This study |
| pCKO1-*tpiAE55.1* | All internal TEV-tag insertions were constructed by amplification and re-ligation of pCKO1-*tpiA* using the forward and reverse primers for each specific insertion variant given in Supplementary Table 5. All reverse primers were purchased as 5'-phosphorilated oligonucleotides. | This study |
| pCKO1-*tpiAE55.2* | See pCKO1-*tpiAE55.1* | This study |
| pCKO1-*tpiAE55.3* | See pCKO1-*tpiAE55.1* | This study |
| pCKO1-*tpiA*N69 | See pCKO1-*tpiAE55.1* | This study |
| pCKO1-*tpiA*T130.1 | See pCKO1-*tpiAE55.1* | This study |
| pCKO1-*tpiA*T130.2 | See pCKO1-*tpiAE55.1* | This study |
| pCKO1-*tpiA*T153.1 | See pCKO1-*tpiAE55.1* | This study |
| pSEVA132-*tpiA*L70 | Constructed as pCKO1-*tpiAE55.1*, but Gibson assembly was used instead of re-ligation. | This study |
| **Adk cleavage analysis** | | |
| pKTs-*adk*wt | pKTs encoding the natural Strep-tagged Adk under control of an anhydrotetracycline inducible promoter (Ptet) | This study |
| pKTs-*adk*76.2 | pKTs encoding Strep-tagged Adk 76.2 (TEV-tag only) under control of an anhydrotetracycline inducible promoter (Ptet) | This study |
| pKTs-*adk*76.3 | pKTs encoding Strep-tagged Adk 76.3 (TEV-tag plus 5 aa flanks at C-terminus) under control of an anhydrotetracycline inducible promoter (Ptet) | This study |
| pKTs-*adk*76.4 | pKTs encoding Strep-tagged Adk 76.4 (TEV-tag plus 7 aa flanks at C-terminus) under control of an anhydrotetracycline inducible promoter (Ptet) | This study |
|  |  |  |
| **TEV protease expression** | | This study |
| pKTs-TEVopt | pKTs encoding for TEVopt (see the section on TEV protease in the Online Methods) under control of a T7 promoter. The protein was produced in strain BL21(DE3) The gene coding for TEVopt was cloned into pKTs via the unique restriction sites *NdeI* and *Xho*I. | This study |
| pEXP3-TEVsol | His-tagged TEV protease (same mutations and deletions as TEVopt) under control of a T7 promoter, expressed as auto-cleaved MBP fusion protein, p15A origin, KanR | Gift from Luzi Pestalozzi |

1. Silva-Rocha R, Martinez-Garcia E, Calles B, Chavarria M, Arce-Rodriguez A, de Las Heras A, Paez-Espino AD, Durante-Rodriguez G, Kim J, Nikel PI *et al*: **The Standard European Vector Architecture (SEVA): a coherent platform for the analysis and deployment of complex prokaryotic phenotypes**. *Nucleic acids research* 2013, **41**(Database issue):D666-675.

2. Fernandez S, Delorenzo V, Perezmartin J: **Activation of the Transcriptional Regulator Xylr of Pseudomonas-Putida by Release of Repression between Functional Domains**. *Mol Microbiol* 1995, **16**(2):205-213.

3. Neuenschwander M, Butz M, Heintz C, Kast P, Hilvert D: **A simple selection strategy for evolving highly efficient enzymes**. *Nature biotechnology* 2007, **25**(10):1145-1147.

4. Billerbeck S, Panke S: **A genetic replacement system for selection-based engineering of essential proteins**. *Microb Cell Fact* 2012, **11**(1):110.
