# Supplementary material for: Sequence-based prediction of permissive stretches for internal protein tagging and knockdown
Source: BMC Biol. 2017 Oct 30;15:100. doi: 10.1186/s12915-017-0440-0 (PMC5661948; doi:10.1186/s12915-017-0440-0)
Supplement: Supplementary file 17 — Primers used in this study. (DOCX 164 kb) [file 12915_2017_440_MOESM17_ESM.docx]

| **Name** | | **Sequence (‘5 to ’3)** |
| --- | --- | --- |
| **TpiA insertions** | | |
| TpiAE55.1-fw | GGCGGTTCTGGTGAAAACCTGTATTTTCAGGGCTCTGGTGGCTCTGGCAGCCACATCATGCTGGG | |
| TpiAE55.2-fw | GGGTGTCTGGGGGAATCTGAAAACCTGTATTTTCAGGGCGATGAAAGAAAGAACAAAGGCAGCCACATCATGCTG | |
| TpiAE55.3-fw | GGCTGCCTGCCGACAACCGAAAACCTGTATTTTCAGAGCGGCACAGTGAAAAACAAAGGCAGCCACATCATGCTG | |
| TpiAE55-rv | TTC AGC TTC GCG CTT CGC CAT ATC | |
| TpiAN69-fw | GAGAATCTTTACTTCCAAGGTCTGTCCGGCGCATTCACCG | |
| TpiAN69-rv | GTT CAG GTC CAC GTT TTG CGC AC | |
| TpiAT130-fw | GAAAACCTGTACTTCCAGGGTGGTTCTGGTAAAACTGAAGAAGTTTGCGCACGTC | |
| TpiAT130.1-rv | GGTTTCACCGATGCACAGAACCG | |
| TpiAT130.2-rv | AGAACCACCGGTTTCACCGATGCACAGAACCG | |
| TpiAT152-fw | GACTATGACATCCCGACAACCGAAAACCTGTATTTTCAGAGCGGCACAGTGGACGCCGGCGCCGACCAGGGTGCTGCGGCATTCGAAG | |
| TpiAT152-rv | AGTTTTCAGTACCGCGTCGATCTG | |
| TpiAL70-fw | GACTCACCTTGGAAGTACAGATTTTCATTTTTCGGTGGCAGGTTCAGGTCCACGTTTTGC | |
| TpiAL70-rv | tgAAAATCTGTACTTCCAAGGTGAGTCcCTGtttaaaggTccaTCCGGCGCATTCACCGG | |
| **Adk insertions** | | |
| *adk*-forward | GGATCCCGGGCCGCAAATTATCTCGCCATTAACCG | |
| *adk*-reverse | CGGAGCTCTTAATTAACGGCCGGCTTAGTGGTGGTGGTGGTG | |
| D76-fw | GAAAACCTGTATTTTCAGGGCAATGGTTTCCTGTTGGACGGCTTC | |
| D76-rv | GTCTTCCTGAGCAATGCGCTC | |
| PSII-library1-fw | GAAAACCTGTATTTTCAGGGGGAAGGGCATCAATGTTGATTACGTTTAATAATTCG | |
| PSII-library2-fw | GAAAACCTGTATTTTCAGGGGGAAGCGGGCATCAATGTTGATTAC | |
| PSII-library3-fw | GAAAACCTGTATTTTCAGGGG AAAGAAGCGGGCATCAATGTTGATTACG | |
| PSII-library4-fw | GAAAACCTGTATTTTCAGGGGGAAGATGAAAGAAGCGGGCATCAATGTTGATTAC | |
| PSII-library5-fw | GAAAACCTGTATTTTCAGGGGGCGATGAAAGAAGCGGGCATCAATG | |
| PSII-library6-fw | GAAAACCTGTATTTTCAGGGGGAAGGACGCGATGAAAGAAGCGGGCATCAATG | |
| PSII-library1-rv | CGCTTCTTTCATCGCGTCTGCCTG | |
| PSII-library2-rv | TTTCATCGCGTCTGCCTGCG | |
| PSII-library3-rv | CATCGCGTCTGCCTGCGGAATGG | |
| PSII-library4-rv | CGCGTCTGCCTGCGGAATG | |
| PSII-library5-rv | GTCTGCCTGCGGAATGGTACGCGG | |
| PSII-library6-rv | TGCCTGCGGAATGGTACGCGGGAAGCCG | |
| AdkP140-fw | GAAAACCTGTATTTTCAGGGGGACGACGTTACCGGTGAAGAAC | |
| AdkP140-rv | CGGCGGATTGAATTTAACGTGAT | |
| AdkA186-fw | GAAAACCTGTATTTTCAGGGGAATACCAAATACGCGAAAGTTGAC | |
| AdkA186-rv | TTCTGCTTCTTTGGAGTAGTAGCC | |
| **GpsA insertions** | | |
| secBgpsA-forward | CGCCCGGGCCATGGGTGTGAACGTTGGCATTACATTGCG | |
| secBgpsA-reverse | GTCTCTAGATTACTTAGTGGTGGTGGTGGTGGTGGTGGCTGCTGCGCTCGTCC | |
| GpsAC49-fw | GAGAATCTGTACTTCCAAGGCTTTCCCGATACGCTCCATCTTG | |
| GpsAC49-rv | ACAGCGGTCGCGTTCAAGC | |
| GpsAP60-fw | GAGAATCTGTACTTCCAAGGCGTGCCTTTTCCCGATACGCTCC | |
| GpsAP60-rv | GGG AAA AGG CAC ATC GGG GAG AAA C | |
| GpsAM99-fw | CCAACCACCGAAAACCTGTATTTTCAGGGGTGTCTGGGGCGTCCTGATGCGCGTCTGG | |
| GpsAM99-rv | CATCAGTGGTTTAATCTGGCGCAG | |
| GpsAI132-fw | CCAACCACCGAAAACCTGTATTTTCAGGGGTGTCTGGGGCCGCTGGCGGTTATCTCTGG | |
| GpsAI132-rv | AATTTGATCGCCTAAGGCCTCACG | |
| GpsAQ269-fw | CCGACCACAGAGAATCTGTACTTCCAAGGCGGCACCGTTGGCATGGATGTACAAAGCGCG | |
| GpsAQ269-rv | CTGACCGAGCATCATGCCAAAAC | |
| GpsAD56.1-fw | CCGATgaaaacctgtattttcagggtGTGCCTTTTCCCGATACGCTC | |
| GpsAD56.1-rv | TATCGGGAAAAGGCACACCCTGAAAATACAGGTTTTCATCGGGGAGAAACGCGGCGTTAC | |
| GpsAD56.2-fw | ctgtattttcagggtgaaagcctgtttaaaggcccgGTGCCTTTTCCCGATACGCTC | |
| GpsAD56.2-rv | ACAGGCTTTCACCCTGAAAATACAGGTTTTCGTTTTTCGGCGGATCGGGGAGAAACGCGG | |
| **pKTS adk** |  | |
| Adk_wt_fw | GCGCATTGCTCA | |
| Adk_wt_rv | AGGAAACCATTACGGCAGTCTTCCTGAGCAATGCGC | |
| Adk76-1-rv | TTTAAACAGGCTTTCCCCCTGGAAGTACAGATTCTCGTCTTCCTGAGCAATGCGCTC | |
| Adk76-1-fw | GAGAATCTGTACTTCCAGGGGGAAAGCCTGTTTAAATGCCGTAATGGTTTCCTGTTG | |
| Adk76-2-fw | GGTCCATGCCGTAATGGTTTCCTGTTGGACGGCTTCCCG | |
| Adk76-2-rv | GAAACCATTACGGCATGGACCTTTAAACAGGCTTTCCCC | |
| **Colony PCR and sequencing for MAGE** | | |
| MutS(mut)-fw [1] | AACCGGACATAA | |
| MutS(wt)-fw [1] | AACCGGACATAACCCCATG | |
| MutS-rv [1] | CGGGATCGGCTCTCC | |
| MutS-seq [1] | GCTGCAAAACAGCATCTTTCC | |
| TEV-rv | CCCCTGAAAATACAGGTTTTC | |
| TEV-2-rv | ACCTTGAAAGTACAAATTCTC | |
| TEV-3-rv | CCCCTGAAAATACAGATTCTC | |
| TEV-4-rv | CCCCTGGAAGTACAGATTCTC | |
| Adk-fw | AAAGGGACTCAGGCTC | |
| Adk-ins2-rv | ACGGCATTTAAACAGGCTTTC | |
| AtpD-fw | GGCATGGGGGATAAC | |
| AtpA-fw | ATGAAGCTGCTAACAGC | |
| AtpA-seq_rv | ACCTTGCCGAAGGCATGAAAG | |
| AtpD-seq_rv | GGATGTAAAAGACCTCGAACAC | |
| Adk-seq-rv | TTAGCCGAGGATTTTTTCC | |
| TpiA-rv | CTGCTCTTTCAGCACCGCG | |
| TpiA-ins1-fw | ATGCGACATCCTTTAGTGATGG | |
| TpiA-ins2-fw | CGGACCCCTGGAAGTACAGATT | |
| TpiA-ins3-fw | ACCTTTAAACAGGGACTC | |
| TpiA-STOP-fw | CATCCTTTAGTGATGGGTtAgTGat | |
| TpiA-STOP-rv | ATAACCGCACCTTCGAATGCCGCAG | |
| Amn-seq-rv | TTTGCCGGGAACTGGCATAG | |
| Amn-STOP-rv | GCTCGGAATAGGAATATCGG | |
| Amn-STOP-fw | AACTCGACGCGCTGTAATAGTG | |
| **MAGE Oligos^a^** | | |
| mutS_off [1] | A*T*C *A*CACCCCATTTAATATCAGGGAACCGGACATAACCCCATCAGTGCAATAGAAAATTTCGA CGCCCATACGCCCATGAT | |
| mutS_on [1] | A*T*C *A*CACCCCATTTAATATCAGGGAACCGGACATAACCCCATGAGTGCAATAGAAAATTTCGA CGCCCATACGCCCATGAT | |
| rpsL_off [2] | G*T*C *A*GACGAACACGGCATACTTTACGCAGCGCGGAGTTCGGTTTTTTAGGAGTGGTAGTATA TACACGAGTACATACGCCACGTTTTTGC | |
| rpsL_on [2] | G*T*C *A*GACGAACACGGCATACTTTACGCAGCGCGGAGTTCGGTTTTCTAGGAGTGGTAGTATA TACACGAGTACATACGCCACGTTTTTGC | |
| bla_off [1] | G*C*C *A*CATAGCAGAACTTTAAAAGTGCTCATCATTGGAAAACGTTATTAGGGGCGAAAACTCT CAAGGATCTTACCGCTGTTGAGATCCAG | |
| bla_on [1] | G*C*C*A*CATAGCAGAACTTTAAAAGTGCTCATCATTGGAAAACGTTCTTCGGGGCGAAAACTCTCAAGGATCTTACCGCTGTTGAGATCCAG | |
| malk_off [2] | C*C*AAATGACATGTTTTCTGCTACTGACAGGTGGGGATAGAGCGCCTAAGACTGAAACACCATACCAACGCCGCGTTCTGCTGGCGGAG*T*G | |
| malK on [2] | C*C*A*A*ATGACATGTTTTCTGCTACTGACAGGTGGGGATAGAGCGCGTAAGACTGAAACACCATACCAACGCCGCGTTCTGCTGGCGGAGTG | |
| atpA_H123 | C*C*G*G*AGCGATTGCTTCTACAGCAGAGAAGCCGTCCCCCTGAAAATACAGGTTTTCGTGATCCAGCGGACCTTTACCGTCGATTGGTGCAC | |
| atpD_E101 | C*G*C*G*GTGAATCGCCCAACGCTCTTCTTCACCGATCCCCTGAAAATACAGGTTTTCCTCGCCTTTCATGTCGACCGGTTCACCCAGTACGT | |
| tpiA_l70_1 | A*C*A*T*CATGCTGGGTGCGCAAAACGTGGACCTGAACCTGCCaCCGAAAAAtGAGGGGTCCGGCGCATTCACCGGTGAAACCTCTGCTGCTA | |
| tpiA_l70_2 | G*C*T*G*GGTGCGCAAAACGTGGACCTGAACCTGCCACCGAAAAATGAGAATCTGTACTTCGGGTCCGGCGCATTCACCGGTGAAACCTCTGC | |
| tpiA_I70_3 | G*C*C*A*CCGAAAAAtGAGAATCTGTACTTCCAGGGGGAGTCcCTGtttaaaggTccaTCCGGCGCATTCACCGGTGAAACCTCTGCTGCTAT | |
| adk_D76 | G*G*A*A*GCCGTCCAACAGGAAACCATTACGGCACCCCTGAAAATACAGATTCTCGTCTTCCTGAGCAATGCGCTCTTTAACCAGCGCGATCA | |
| adk_D76_2 | C*G*C*G*GGAAGCCGTCCAACAGGAAACCATTACGGCATTTAAACAGGCTTTCCCCCTGAAAATACAGATTCTCGTCTTCCTGAGCAATGCGC | |

1. Carr PA, Wang HH, Sterling B, Isaacs FJ, Lajoie MJ, Xu G, Church GM, Jacobson JM: **Enhanced multiplex genome engineering through co-operative oligonucleotide co-selection**. *Nucleic acids research* 2012, **40**(17).

2. Nyerges A, Csorgo B, Nagy I, Latinovics D, Szamecz B, Posfai G, Pal C: **Conditional DNA repair mutants enable highly precise genome engineering**. *Nucleic acids research* 2014, **42**(8).
